# A review of prospective pathways and impacts of COVID-19 on the accessibility, safety, quality, and affordability of essential medicines and vaccines for universal health coverage in Africa

**DOI:** 10.1186/s12992-021-00666-8

**Published:** 2021-04-08

**Authors:** Floriano Amimo, Ben Lambert, Anthony Magit, Masahiro Hashizume

**Affiliations:** 1 Department of Global Health Policy, Graduate School of Medicine, The University of Tokyo, Tokyo, Japan; 2 Faculty of Medicine, Eduardo Mondlane University, Maputo, Mozambique; 3 MRC Centre for Global Infectious Disease Analysis, School of Public Health, Imperial College London, London, W2 1PG UK; 4 Human Research Protection Program, University of California San Diego School of Medicine, San Diego, California USA

**Keywords:** COVID-19, SARS-CoV-2, Health systems, Essential medicines, Vaccines, Universal health coverage, Health policy, Africa

## Abstract

**Background:**

The ongoing pandemic of coronavirus disease 2019 (COVID-19) has the potential to reverse progress towards global targets. This study examines the risks that the COVID-19 pandemic poses to equitable access to essential medicines and vaccines (EMV) for universal health coverage in Africa.

**Methods:**

We searched medical databases and grey literature up to 2 October 2020 for studies reporting data on prospective pathways and innovative strategies relevant for the assessment and management of the emerging risks in accessibility, safety, quality, and affordability of EMV in the context of the COVID-19 pandemic. We used the resulting pool of evidence to support our analysis and to draw policy recommendations to mitigate the emerging risks and improve preparedness for future crises.

**Results:**

Of the 310 records screened, 134 were included in the analysis. We found that the disruption of the international system affects more immediately the capability of low- and middle-income countries to acquire the basket of EMV. The COVID-19 pandemic may facilitate dishonesty and fraud, increasing the propensity of patients to take substandard and falsified drugs. Strategic regional cooperation in the form of joint tenders and contract awarding, joint price negotiation and supplier selection, as well as joint market research, monitoring, and evaluation could improve the supply, affordability, quality, and safety of EMV. Sustainable health financing along with international technology transfer and substantial investment in research and development are needed to minimize the vulnerability of African countries arising from their dependence on imported EMV. To ensure equitable access, community-based strategies such as mobile clinics as well as fees exemptions for vulnerable and under-served segments of society might need to be considered. Strategies such as task delegation and telephone triage could help reduce physician workload. This coupled with payments of risk allowance to frontline healthcare workers and health-literate healthcare organization might improve the appropriate use of EMV.

**Conclusions:**

Innovative and sustainable strategies informed by comparative risk assessment are increasingly needed to ensure that local economic, social, demographic, and epidemiological risks and potentials are accounted for in the national COVID-19 responses.

**Supplementary Information:**

The online version contains supplementary material available at 10.1186/s12992-021-00666-8.

## Background

The current coronavirus disease 2019 (COVID-19) pandemic is a global public health emergency that requires extraordinary measures to control. This is warranted because of its high transmissibility and capacity to disrupt international travel and business, among other factors [1, 2]. However, other major causes of death and suffering (MCDS), some of which once also created travel and business restrictions and were responsible for catastrophic losses of lives and suffering in Africa and elsewhere, are still rampant [3–11]. These include human immunodeficiency virus infection and acquired immune deficiency syndrome (HIV/AIDS), tuberculosis, malaria, cholera, polio, measles, meningitis, among others. HIV/AIDS is the leading cause of death across the continent. From 1990 to 2017 the number, rate, and share of HIV/AIDS deaths increased by 156.0, 22.6, and 132.0%, respectively in sub-Saharan Africa (SSA) [4]. Currently, approximately 20.7 million (uncertainty interval: 18.4–23.0 million), and 4.9 million (3.9–6.2 million) people live with HIV/AIDS, in eastern and southern Africa, and in western and central Africa, respectively [9]. With approximately 13.7% of the global population [7], the World Health Organization (WHO) African region accounted for 93% of total malaria cases and 94% of global malaria deaths in 2018 [6]. Moreover, > 6000 attributable deaths and > 310,000 suspected cases of measles were reported in the Democratic Republic of the Congo (DRC) in 2019 [3, 11]. This is despite the availability of MMR (or MMRV for measles, mumps, rubella, and varicella) vaccine that is known to be 93 and 97% effective against measles with one and two doses, respectively [12]. Nevertheless, important health gains have been accomplished in many key indicators in the last two decades.

To preserve and scale up the health gains accomplished so far, responding adequately to emerging (e.g., COVID-19 pandemic [2]) and re-emerging (e.g., measles outbreak [3, 11], increasing HIV/AIDS deaths [4], Ebola outbreak [11, 13]) infectious diseases, to achieve global targets (e.g., Sustainable Development Goals target 3.8), a stable and resilient supply of essential medicines and vaccines (EMV) in the face of global crises and national vulnerabilities is critical. Most low-income countries (LIC) rely on official development assistance (ODA), multilateral support, and the global supply chain (GSC) to acquire EMV and to implement the actions needed to achieve the global targets. However, some institutions that helped accomplish unprecedented gains in relation to these diseases are now focusing only on COVID-19 [14]. This tendency coupled with the disruption of the GSC might create important pressure on national finances and health systems of LIC and risks reversing important health gains.

Modelling studies have shown that even if the pandemic is controlled as a result of current primarily non-pharmaceutical measures, subsequent waves of severe acute respiratory syndrome coronavirus 2 (SARS-CoV-2) infections might occur when these measures are relaxed [2, 15, 16]. Moreover, the large number of deaths due to the measles outbreak—for which there is an effective vaccine—reported in the DRC is an indication that a COVID-19 vaccine and/or cure might not be the panacea for COVID-19, at least in many LIC. Therefore, it is vital to ensure the mid- and long-term feasibility of national COVID-19 responses to minimize their anticipated detrimental societal impacts [17–33] and to prevent the emergence and/or escalation of further risks, particularly among the most vulnerable economic and health systems.

In this study, we examine how the COVID-19 pandemic might affect the accessibility, safety, quality, and affordability (ASQA) of EMV for universal health coverage (UHC) in Africa. We use current evidence retrieved from the literature to support our analysis and to draw policy recommendations to mitigate the emerging risks.

## Methods

### Search strategy

We conducted an extensive search of databases using the algorithm detailed in Table 1. We searched in LitCovid, Scopus, MEDLINE (OVID), CINAHL (EBSCO), Web of Science Core Collection, African Index Medicus, Cochrane Library, WHO databases, preprint servers, and Google Scholar. We additionally consulted the Global Health Observatory data repository as well as databases of regional intergovernmental organizations and economic communities and international financial and global health organizations for relevant data. We performed citation backtracking to identify additional sources. The search for evidence was done from 22 April 2020 to 2 October 2020 by the lead author.

**Table 1:** Search algorithm applied to medical databases to retrieve evidence on prospective pathways and innovative strategies relevant for the assessment and management of the emerging risks in accessibility, safety, quality, and affordability of essential medicines and vaccines in the context of the COVID-19 pandemic. The algorithm was applied to LitCovid, Scopus, MEDLINE (OVID), CINAHL (EBSCO), Web of Science Core Collection, African Index Medicus, and Cochrane Library. Evidence gathering was done from April 22 to October 2, 2020

Module	Set	Terms
I. Assessment of the potential/observed pathways and impacts of COVID-19 on service delivery and/or equitable access to quality medicines and vaccines	1	“COVID-19” or “SARS-CoV-2”
	2	“healthcare” or “medicines” or “drugs” or “vaccines” or “service”
	3	“quality” or “access” or “cost” or “affordability” or “equity” or “coverage” or “safety” or “prescription” or “innovation”
	4	“Africa”
	5	1 and 2 and 3 and 4
II. Innovations in service delivery and/or management and supply of essential medicines and vaccines applicable to manage the risks potentially associated with the COVID-19 pandemic	1	“drug manufacturing” or “drug procurement” or “mobile clinics” or “telephone triage” or “user fees exemption” or “task delegation” or “risk allowance” or “online medical education”
	2	“effectiveness” or “cost-effective” or “efficiency” or “outcomes” or “strategies” or “innovations”
	3	“Africa”
	4	1 and 2 and 3

### Eligibility assessment

**Fig. 1:**
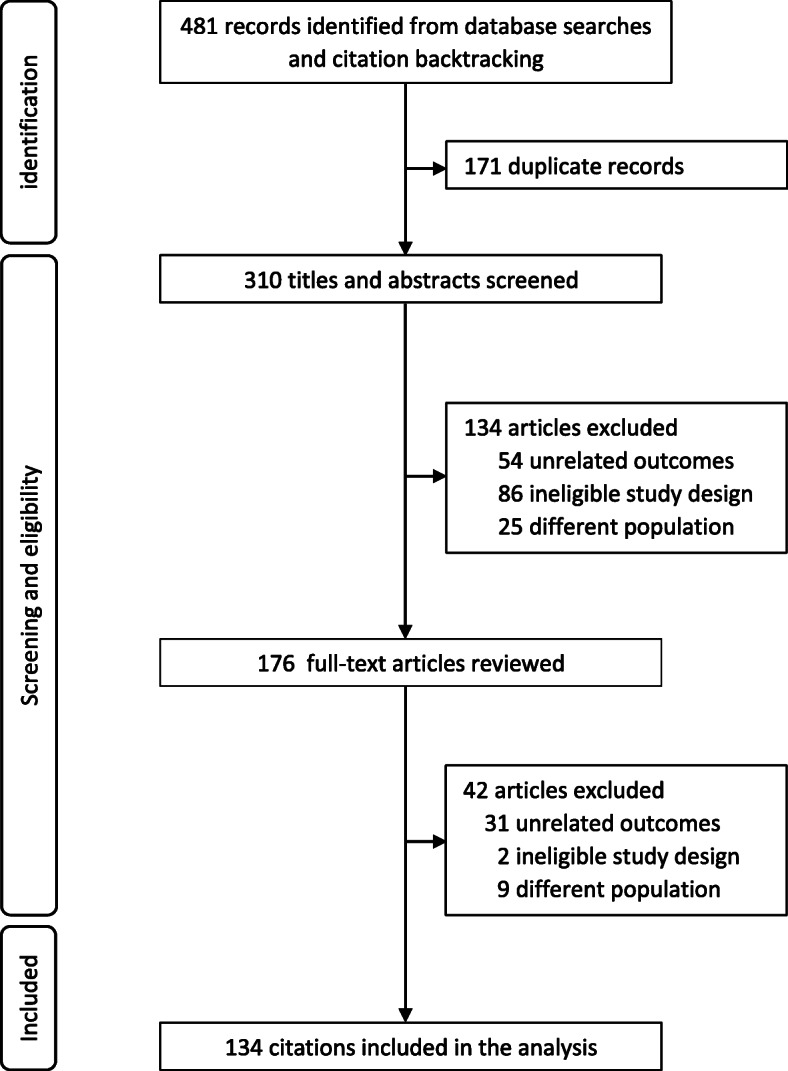
PRISMA flowchart illustrating eligibility assessment of studies reporting data relevant for the assessment and management of the prospective pathways and impacts of COVID-19 on the accessibility, safety, quality, and affordability of essential medicines and vaccines for universal health coverage in Africa. Our search strategy yielded the following numbers of records per database/source (data are shown as: [records for module 1]/[records for module 2] [name of database/source]): 85/1 LitCovid, 115/51 Scopus, 52/24 MEDLINE (OVID), 7/4 CINAHL (EBSCO), 27/22 Web of Science Core Collection, 5/0 African Index Medicus, 2/1 Cochrane Library, and 54/31 other sources. Evidence gathering was done from April 22 to October 2, 2020. The sum of titles and abstracts excluded by each reason > total titles and abstracts excluded. This is because titles and abstracts were excluded by not meeting several eligibility criteria

Eligibility assessment was done following PRISMA (Preferred Reporting Items for Systematic Reviews and Meta-Analyses) process flow (Fig. 1). We included original studies, reviews, and analyses reporting data on prospective pathways and innovative strategies relevant for the assessment and management of the emerging risks in equitable access to quality EMV in the context of the COVID-19 pandemic, published in English up to 2 October 2020, and whose full text is available. For evidence pertaining to module 1, we excluded records that did not report data applicable to the assessment of the potential pathways or impacts of the COVID-19 pandemic on the ASQA of EMV in low- and middle-income countries (LMIC). Nevertheless, to improve our evidence base in relation to module 1 we additionally consulted studies conducted in high-income countries (HIC) involving under-served segments of society. For evidence pertaining to module 2, we excluded records that did not report data on the applicability, affordability, cost-effectiveness, or health outcomes of the discussed innovations in service delivery (SD) and management and supply of EMV in LMIC (Table 1). Eligibility assessment was done by FA and validated independently by BL, AM, MH. Records were collated and reviewed using Endnote X8.

### Analysis

From each eligible study, we extracted data on (a) study design, period, and publication year/venue, (b) geographies and populations covered, (c) pathways by which COVID-19 can impact equitable access to, or supply of, quality EMV, (d) innovative SD models/solutions and/or innovative supply management strategies that can be applied to manage the risks posed by the COVID-19 pandemic, and (e) the applicability, affordability, cost-effectiveness, and/or health outcomes of the pathways/models/strategies in LMIC. Data were extracted by the lead author and validated independently by BL, AM, MH. Using the resulting pool of evidence, we subsequently employed the framework outlined by The Lancet’s Commission on Essential Medicines Policies [34] to identify the major risks posed or escalated by the current crisis and to draw comprehensive policy recommendations on how to mitigate them and improve preparedness for future crises. The domains of the framework are: (1) paying for a basket of essential medicines; (2) making essential medicines and vaccines affordable; (3) assuring the quality and safety of medicines to prevent harm to patients; (4) promoting quality use of essential medicines to ensure better health outcomes; (5) the need for global research and policy framework to develop missing essential medicines. To address comprehensively the problem under study, we expanded the coverage of the first dimension of the analytical framework to include financing, purchasing, and logistics. This was done because the data fits this expansion inductively [35]. The analysis was conducted by FA, who drafted the assignment matrix by matching the data from each eligible study with each domain of the analytical framework across modules. The assignment matrix was subsequently shared with and validated independently by each co-author. Following the WHO, we define EMV as drugs that satisfy the priority healthcare needs of the population, selected according to disease prevalence, public health relevance, clinical efficacy and safety, and comparative costs-effectiveness [36]. Our assessment is applicable to any COVID-19 cure and/or vaccine.

## Results

We identified 310 unique records from database searches and citation backtracking. Of these, 134 records were excluded during title-abstract eligibility assessment. Therefore, 176 full texts were reviewed, of which 42 were excluded, as illustrated in Fig. 1. As a result, 134 citations were included in the analysis of the prospective pathways and implications of the COVID-19 pandemic on the ASQA of EMV for UHC in Africa and in the formulation of applicable policy recommendations to mitigate the emerging risks. The resulting pool of evidence across domains and modules is outlined in Supplement 1. The major risks posed/escalated by the COVID-19 pandemic on the equitable access to EMV, along with the recommendations and stakeholders relevant for issues pertaining to each domain, are detailed in Table 2.

**Table 2:** Major risks posed/escalated by the COVID-19 pandemic on the equitable access to essential medicines and vaccines for universal health coverage, along with the recommendations and stakeholders relevant for issues pertaining to each area. Regional intergovernmental organizations and economic communities include: African Union, Southern African Development Community, Common Market for Eastern and Southern Africa, East African Community, Economic Community of Central African States, Economic Community of West African States, Intergovernmental Authority on Development, Community of Sahel–Saharan States, and Arab Maghreb Union

Area	Risks	Recommendations	Stakeholders
Assuring the financing and supply of essential medicines and vaccines	-Poor financial capability of national health and economic systems to purchase the basic basket of essential medicines and vaccines -Disruption of global supply network due to COVID-19 -International partners focusing only on COVID-19 at the expenses of other major diseases and internally	-Resource allocation informed by CRA -Include sustainable financing and stable supply of EMV in the workstreams of COVID-19 supply task forces -Integrate logistics system of COVID-19 with those of other major diseases -Joint tenders and contract awarding at regional level -Joint price negotiation and supplier selection at regional level -Joint market research, monitoring, and evaluation at regional level -Strategic procurement and regional information sharing about suppliers and prices	-World Health Organization -Regional intergovernmental organizations and economic communities -National governments -Nongovernment organizations -Private sector
Making essential medicines and vaccines affordable	-Reduced availability of suppliers -Reduced capacity of patients to purchase health services because most people in the productive age work in the informal sector, with household income based mainly on daily earning -Unavailability of transportation and/or long distance to health facilities -Inefficiency or inexistence of effective social protection programmes	-Mobile clinics -Fees exemption for under-served and vulnerable segments of society -Microfinance loans (with transparent selection of participants informed by literature of predictors of repayment rate) -CHW programmes (conditional on significant increase in ODA and/or important innovations in health financing) -Multi-month dispensing of essential drugs for selected diseases -Integrated service delivery	-National and subnational governments -Private sector -Nongovernment organizations
Assuring the quality and safety of medicines to prevent harm to patients	-Limited human and technical capability -Poor transparency in procurement of essential drugs (e.g., lack of competitive bidding, corruption embedded in health systems) -Restrictions to national and international business and travel imposed as part of national COVID-19 responses	-Investment in good governance at global, national, and local levels -Investment in sustainable financing at global, national, and local levels -Strategic investment in human and technical capacity building -Joint market research, monitoring, and evaluation at regional level -Regional information sharing about suppliers and prices -Medicines regulatory harmonization at regional level -International cooperation to equip LMIC with the necessary technology for post-marketing surveillance of medicines	-United Nations Development Programme -International and national financial institutions -World Health Organization -National governments -Regional intergovernmental organizations and economic communities -International and national financial institutions
Promoting quality use of essential medicines to ensure better health outcomes	-Increased workload per HCW because of change in work modality as part of COVID-19 measures -Reduced quality of HCW because of risks to health work force regeneration processes -Reduced capacity of the health sector to make payments -Poor health literacy and numeracy of patients -Limited health-literate healthcare organization	-Task delegation -Telephone triage -Sustainable international exchange of HCW targeting jointly delivery of clinical services (immediate needs) and capacity building of LMIC to generate quality health workforce efficiently (improve preparedness) -Payment of risk allowances to frontline HCW -Investment in health literacy and numeracy of populations and patients -Careful staff recruitment -Detailed training of HCW -Authorization to provide autonomous care -Reliable data systems -Fair and performance-based compensation of HCW	-National governments -Bilateral partners
The need for global research and policy framework to develop missing essential medicines	-Limited availability of high-level human, technological, and financial capability -Patents restrictions -Low investment in R&D -Poor medicines regulatory capacity	-Substantial increase in investment in R&D capability -Sustainable technology transfer and international financing -Strategic investment in human and technical capacity building -Facilitate cross-border trade of pharmaceutical goods in the region	-National governments -United Nations Industrial Development Organization -World Trade Organization -Regional intergovernmental organizations and economic communities -Private sector -Universities and research institutions -International and national financial institutions

*CHW* = community health worker, *COVID-19* = coronavirus disease 2019, *CRA* = comparative risk assessment, *EMV* = essential medicines and vaccines, *HCW* = healthcare workers, *LMIC* = low- and middle-income countries, *ODA* = official development assistance, *R&D* = research and development

## Discussion

### Assuring the financing and supply of essential medicines and vaccines

Many LMIC spend less than the $13 to $25 per capita required to purchase a basic package of 201 EMV [34]. Therefore, to promote sustainable access for all and reduce out-of-pocket spending, among other recommendations, The Lancet’s Commission on Essential Medicines Policies urged the international community (IC) to support governments of LIC in financing a basic package of EMV. We identified two major risks in relation to this area. Firstly, the COVID-19 pandemic further deteriorates the fragile capability of African countries to finance the health sector. On the one hand, plummeting oil prices and a lowered global demand for African non-oil products threatens the economic stability of many countries across the continent [37]. It is estimated that the pandemic might lead to revenues from fuel exports falling to around $101 billion in 2020 on the continent, representing a decline of $65 billion compared to average 2016–18 yearly exports revenues of $166 billion [19]. On the other hand, there is a tendency of some international partners to focus only on addressing COVID-19. For instance, Bill & Melinda Gates Foundation has announced that the organization will now focus entirely on COVID-19 [14]. Additionally, in many countries, ODA is tied to gross national income (GNI); therefore, due to the expected contraction of many advanced economies, some bilateral partners (BP) might make significant reductions in the funding of projects linked to ODA [20, 38]. For instance, no new resources have been allocated to President’s Emergency Plan for AIDS Relief (PEPFAR), other than emergency funding for certain global efforts and to domestic HIV programmes [39]. This could significantly affect the financing of the health sector and consequently the financial capability of national governments to purchase the basket of EMV across the continent. Furthermore, an increase in the cost of production and distribution of EMV is expected because of the lockdowns and border closures. For instance, the final cost to export antiretroviral drugs (ARV) from India is estimated to be 10 to 25% higher compared to prices before the COVID-19 pandemic [9]. Even with the financing secured, the procurement of EMV could also be impacted negatively by the disruption of the GSC and closure/restrictions of external borders. This might also affect the supply of insecticide-treated bed nets (ITN) for malaria, which are of comparable importance to EMV in malaria-endemic countries.

The WHO in collaboration with the World Food Programme has set up a supply task force to ensure that the medicines and equipment needed to respond to COVID-19 are provided to resource-constrained settings. This task force is a high-level supervisory body which convenes key partners and provides strategic direction and guidance to ensure effective functioning of the COVID-19 Supply Chain System [35]. In Africa, the African Union (AU) Commission, Africa Centres for Disease Control and Prevention (Africa CDC), and WHO have also established another task force termed African Task Force for Coronavirus Preparedness and Response (AFTCOR). In addition to supply chain management, the focus of AFTCOR includes increasing capacity for laboratory diagnostics, surveillance and cross-border screening, infection prevention and control in health facilities, clinical treatment of cases, and risk communication [40]. Both these task forces focus on the supply of drugs and equipment needed to directly manage COVID-19. Likewise, other global organizations have also been directing their efforts to COVID-19. For example, Gavi, the Vaccine Alliance has allocated $200 million to COVID-19 response, with $29 million approved to fund efforts directly targeting the pandemic in 13 LIC (e.g., protective equipment for healthcare workers (HCW), surveillance, and testing) [41]. The rechannelling of international funds and the setting up of regional task forces to focus more on COVID-19 response suggests a further imposition of donor-driven vertical programmes. This has been extensively discussed in the context of HIV/AIDS, tuberculosis, malaria, and other health issues, and typically involves the introduction of parallel systems and processes by global health actors and programme implementers under pressure to deliver short-term results [42]. Therefore, current efforts in the fight against the COVID-19 pandemic in Africa neglect extensive evidence on the detrimental effects of fragmentation in global health on health systems and aid effectiveness in LMIC.

For most advanced and some emerging economies, where most of COVID-19 cases and deaths are concentrated to date, the immediate risks associated with critical supply shortages concern mostly shortage of equipment and drugs needed to directly manage the pandemic. These include ventilators and personal protective equipment [43]. However, for many AU member states, which so far have registered relatively few COVID-19 cases and deaths compared to advanced economies (with a few outliers) [1, 44], the major effect of the disruption of the GSC due to the COVID-19 pandemic concerns mostly shortage of life-saving drugs that are needed to respond to other epidemic diseases that are MCDS across the continent, in addition to medicines and equipment needed to manage COVID-19. Many of these countries already experienced under-stocking and stock-outs of these EMV before COVID-19 [45, 46], and this will be escalated by the current crisis with potentially catastrophic consequences [17–32]. A modelling analysis conducted by the WHO shows that a severe disruption in the supply of ITN and effective antimalarial medicines could double the number of malaria deaths compared to 2018, potentially reversing the trends in malaria deaths to the levels seen 20 years ago (i.e., approximately 769,000 malaria deaths are projected in 2020 in Africa in the worst case scenario—70% of which in children aged < 5 years) [21]. Further estimates show that a six-month interruption of supply of ARV could result in more than 500,000 (471,000–673,000) adult HIV deaths across Africa over 1 year (in addition to the usual HIV deaths, estimated at 470,000 in 2018), while a disruption in preventing mother-to-child transmission of equal length would lead to an increase of perinatally acquired HIV infections of 162, 139, 106 and 83% in Malawi, Uganda, Zimbabwe, and Mozambique, respectively [9, 29]. In comparison, according to Africa CDC, a total of 36,372 COVID-19 deaths have been registered across AU member states as of 2 October 2020 [44], although limited testing and surveillance capabilities may contribute to this figure. Taken together, the data indicate that the major threat of COVID-19 in Africa might not be the disease directly, but rather the associated disruption of the control of other major epidemic diseases on the continent. Yet no similar systems or task forces have been set up to ensure sustainable financing and stable supply of life-saving drugs that countries with vulnerable economic and health systems need to continue delivering essential health services other than the response to COVID-19.

To better respond to COVID-19, tackling the potential detrimental impacts of the pandemic on other MCDS across the continent should be part of the workstreams of these task forces or similar initiatives. Importantly, resource allocation by the IC and national governments in Africa in response to the COVID-19 pandemic should be informed by comparative risk assessment (CRA). This means that the direct burden of COVID-19 under various response scenarios should be systematically compared to changes in population health resulting from other MCDS across the continent under comparable scenarios. Given the high burden of other MCDS across the continent (compared to COVID-19 cases and deaths based on current data) [1, 2, 17, 18, 21, 24, 44, 47] coupled with national vulnerabilities and the disruptive nature of COVID-19 [19, 20, 22, 33], we argue that the COVID-19 supply system should be used to request and deliver EMV as well as ITN, in addition to the medicines and equipment needed to directly manage COVID-19. To minimize the risk of further disrupting the supply of EMV because the COVID-19 supplies platform is in its infancy, priority should initially be given to life-saving drugs with a relatively high propensity to be in short supply, and gradually move towards integration by adding other EMV to the platform. This could partially mitigate the immediate risks associated with potential negative impacts of the pandemic on the capability of vulnerable countries to acquire EMV needed to control other MCDS in the region. Strategic regional cooperation is crucial in the face of global risks and national vulnerabilities. This in the form of joint tenders and contract awarding, joint price negotiation and supplier selection, as well as joint market research, monitoring, and evaluation could help countries across the continent mitigate the current risks and improve their capability to respond to future crises [48].

### Making essential medicines and vaccines affordable

This is critical to achieve equity in access. Affordability is related to direct and indirect costs of EMV. This is influenced by availability of suppliers, competitiveness of the market, capacity of patients to purchase health services, transportation and/or distance to health facilities, and government policies, among other factors [49, 50]. The COVID-19 disruption of the international system might affect the availability of suppliers and the competitiveness of LMIC markets. Moreover, most people in the productive age in the WHO African region work in the informal sector, with household income based mainly on daily earning [18]. Because of the disruptions associated with COVID-19 containment measures (e.g., closure/restriction of educational institutions/activities, ban/restriction of intra-urban public transportation, ban/limitation of inter-urban movements, closure of workplaces/restrictions of business, closure/restrictions of external borders, ban/restrictions of public gatherings/events), many businesses might become unsustainable. Recent projections show that as a result of these measures, an additional 9.1% of the population across SSA are estimated to have fallen into extreme poverty, with 30% of the population across the continent projected (under an eight-week lockdown scenario) to lose their resilience capacity to future shocks [22]. Therefore, many patients are expected to lose their already fragile capacity to purchase medicines or even to reach health facilities. Inefficiency or inexistence of effective social protection programmes constitutes an important challenge to any efforts aiming at mitigating these risks (e.g., an individual in the poorest income quintile typically has a 4% chance to receive social assistance from the government [22]).

To mitigate the risks that the current crisis poses to equitable access to EMV, governments might have to consider policies aimed at protecting under-served and vulnerable segments of society, rather than just copy-pasting COVID-19 strategies from HIC as it is being done currently across Africa and elsewhere in LMIC [51].

Temporarily exempting certain segments of society from user fees payment at public (and potentially private) pharmacies and hospitals could help reduce financial hardship associated with out-of-pocket payments [52–57]. These populations could include people living with HIV/AIDS (PLWHA), patients with high-risk pregnancy, pregnant women living distant from health facility, patients under treatment for tuberculosis, households with sick children, communities living in malaria-endemic settings, among others. However, to adequately improve equitable access to EMV, sustained user fee exemptions for large population groups might be needed [54, 57, 58]. Historically, these charges were levied due to supposed constraints in public financing, which are compounded by declining national incomes in the context of the current pandemic (as indicated above). Therefore, for a successful implementation of fee exemptions, innovative and sustainable health financing models are critical. This includes alignment of government budgets with the Abuja Declaration, by which in 2001 African governments pledged to allocate ≥15% of their annual budget to improve the health sector and urged donor countries to allocate 0.7% of their GNI as ODA to LMIC (a long-standing United Nations target) [59]. Across SSA, most countries (93.5%) have government health spending per general government spending < 15%, except for Namibia (17.8%), South Africa (17.4%), and eSwatini (15.0%), as per 2015 metrics [60]. Likewise, most donor countries (86.4%) spend < 0.7% of GNI as ODA, except for Turkey (1.150%), Luxembourg (1.050%), Norway (1.026%), Sweden (0.956%), Denmark (0.713%), and the United Kingdom (0.702%), as per 2019 metrics [61]. Priority-setting should be informed by CRA.

Community-based innovative approaches, such as mobile clinics (MC), could be used to deliver EMV to those patients facing unprecedented difficulties reaching health facilities due to limited capacity to pay for transportation, long distance to a health facility, and/or unavailability of public transport. Among others, these might include ARV (for both treatment of PLWHA (including trimethoprim-sulfamethoxazole) and prevention of mother-to-child transmission, along with HIV testing and counselling, and condom distribution), antimalarial drugs (for both preventive and curative treatments, along with testing and ITN distribution), childhood vaccines, antituberculosis drugs, iron and folic acid for pregnant women, and contraceptives. It is estimated that 287,282,013 people and 64,495,526 women of childbearing age live > 2 h from the nearest hospital [62]. Therefore, MC could help reduce the cost of, and inequalities in access to, EMV. MC have been shown to be feasible and cost-effective for active tuberculosis case finding, provision of antiretroviral therapy (ART), as well as HIV testing and counselling [63–66]. When applied to ART, MC have been associated with a 10-year mean undiscounted life-expectancy of 4.3 life-years (LY) and mean discounted 2.9 quality-adjusted life-years (QALYs) compared to 3.6 LY and 2.3 QALYs for facility-based delivery [65]. Moreover, MC have been associated with improved health indices of children in resource-limited settings [67]. Integrated SD is vital to ensure cost-effectiveness [68]. This has been raised in the context of other conditions, such as HIV. Despite the widely reported cost-effectiveness [69] and acceptability [70] of MC, some logistical challenges as well as spatial and structural constraints have been documented [71]. Therefore, efforts to ensure confidentiality and privacy of patients as well as full engagement with the community and culturally informed recruitment of HCW are vital for a successful implementation of MC.

Microfinance loans in the form of health saving plans or emergency health loans have the potential to protect vulnerable segments of society from catastrophic health expenditure. However, a recent study examining implementation of this healthcare financing scheme in Tanzania indicated mixed results regarding repayment [72]. Nevertheless, the study also found that repayment rate is positively correlated with group leadership, prior business experience, and training in loan repayment. This suggests that even though this strategy might not be feasible universally as a tool to mitigate the impact of COVID-19 on equitable access to life-saving drugs, it (in conjunction with other strategies to cover other segments of society) could help certain patients carefully selected using criteria that have been shown in the literature to be correlated with high repayment rates. Community health worker (CHW) programmes also have the potential to help improve equitable access to EMV. However, a recent study has indicated that these programmes might not be affordable in many countries across the continent: affordability of CHW programmes seems to decline as gross domestic product (GDP) per capita increases [73]. Therefore, CHW programmes might not be feasible to mitigate the risks posed by COVID-19, without a significant increase in ODA and/or important innovations in health financing. PEPFAR has supported multi-month dispensing of ARV to ensure continuity of care [39]. Although this strategy is applicable to mitigate (at least in part) the potential impact of the pandemic on equitable access to EMV for chronic diseases (e.g., HIV/AIDS), it cannot be used to address most of the major diseases in the region (e.g., malaria).

Without these kinds of measures, summarized in Table 2, the COVID-19 pandemic may result in an unprecedented increase in health inequalities, with catastrophic consequences in health outcomes. While innovative SD models have existed in many countries, including those with a high HIV burden through what is known as differentiated SD models, the emerging risks in the face of the current pandemic highlight the urgency with which these need to be scaled up to protect the most vulnerable segments of society. Importantly, if these risks are addressed using integrated and sustainable solutions, the COVID-19 pandemic could become a catalyst for UHC: helping to ensure equity in access.

### Assuring the quality and safety of medicines to prevent harm to patients

This is a persistent problem in many LMIC, particularly in SSA [74–81]. Limited capability of health systems to perform post-marketing surveillance of medicines has been an important factor contributing to poor quality of drugs across the continent [82, 83]. Previous studies have indicated that even though most drugs pass the tests for pharmaceutical dosage forms, they fail to meet the required pharmacological specifications [84]. Other studies have so far focused more on the risk that substandard and/or falsified tests, drugs, vaccines pose to COVID-19 [85]. Here we show that for Africa, where most countries have so far registered relatively few COVID-19 cases and deaths compared to many advanced economies [1, 44], the major risks are those that the pandemic poses to the supply of quality and safe EMV that are most consumed across Africa. These are drugs that are needed to control other important diseases that are MCDS—and most of them are more fatal than COVID-19—across the continent [3, 4, 8, 13, 21, 24].

Our assessment is that most of the recommendations of The Lancet’s Commission on Essential Medicines Policies for this area might not be feasible across the WHO African region. Limited human and technical capability is the major constraint. Poor transparency in the procurement of EMV (e.g., lack of competitive bidding [86], corruption embedded in health systems [87]) in most of Africa is another important factor. The COVID-19 pandemic might make this worse in many ways. For example, because of a state of emergency/disaster/calamity that has been declared in many countries with flexibility in procuring and purchasing medical products (MP) in view of the COVID-19 crisis, it might make it more challenging for some potential suppliers to compete fairly, and this might facilitate dishonesty and fraud. Therefore, the COVID-19 pandemic might result in an increased propensity of patients to take EMV that are substandard (i.e., authorized MP that fail to meet either their quality standards or their specifications, or both [88]) and/or falsified (i.e., MP that deliberately/fraudulently misrepresent their identity, composition, or source [88]), beyond MP for COVID-19. Moreover, multi-month dispensing of medicines, if not coupled with instructions for safe storage at home, could also contribute to poor quality because drugs might suffer degradation due to poor storage after leaving the pharmacy. For HIV, tuberculosis, and malaria, this might contribute to the emergence and spread of drug resistant pathogens, increase infectiousness of PLWHA and tuberculosis patients, and render the current control measures ineffective [18, 89, 90]. Tuberculosis, which is the leading cause of death among PLWHA, has a case fatality rate of 41.0% among patients with rifampicin-resistant or multidrug-resistant tuberculosis [8].

Therefore, this area poses important risks and challenges as illustrated in Table 2. These might be escalated by the restrictions imposed as part of national COVID-19 responses, with the potential to become the source of larger crises in the region. Investment in good governance and sustainable financing at global, national, and local levels are critical to address the drivers of the risks associated with this area in the mid- and long-term [91–94]. Joint market research, monitoring, and evaluation, coupled with regulatory harmonization at regional levels and sustainable international cooperation to equip LMIC with the necessary technology (e.g., high-performance liquid chromatography, thin layer chromatography, Raman spectroscopy, UV-Vis spectroscopy), might improve the quality of EMV [48, 78, 81, 85, 95–98].

### Promoting quality use of essential medicines to ensure better health outcomes

This is related to HCW: workload, quality, and motivation. Availability of quality equipment, health literacy and numeracy of patients, health-literate healthcare organization, as well as other factors might also contribute [99–104]. As part of the response to COVID-19, many governments across the continent and elsewhere have implemented extraordinary measures to curb the spread of SARS-CoV-2 infection. In this context, HCW are required to work by shift in some countries across the continent (e.g., Mozambique). This might result in an increased workload per HCW. This increased workload has the potential to reduce the capacity of HCW to prescribe drugs correctly [105, 106]. Innovative strategies such as task delegation and telephone triage could help reduce physician workload and thus help improve the quality use of essential medicine prescribing [65, 107–111]. A growing body of evidence indicates that shifting care from doctors to nurses or other HCW with less training and fewer qualification is cost-effective; however, the evidence is mixed in relation to the cost-effectiveness of CHW. A systematic review covering LMIC globally indicated that task-shifting or CHW is likely to be cost-effective compared to doctor-led or health facility-based care [69]. However, a recent study conducted in Africa has indicated that CHW programmes might not be affordable in many countries across the continent because (as indicated above) the affordability of these programmes seems to decline as GDP per capita increases [73].

COVID-19 might affect the quality of HCW in the mid- and long-term (but less likely in the short-term)—mainly by affecting workforce regeneration processes. This is because the efficiency and effectiveness of e-learning in medical education has not been rigorously tested before, particularly in resource-constrained settings [112]. Limited institutional readiness in human and infrastructural resources is the major constraint for effective implementation of online medical education in LMIC, among others [113]. COVID-19 might not directly affect motivation of HCW to prescribe drugs appropriately. However, COVID-19 might affect the capacity of the health sector to make payments, as reported in west Africa during the Ebola outbreak [114]. This includes payment of risk allowances [115]. This has the potential to indirectly compromise HCW motivation, particularly because a significant increase in the prices of basic needs (e.g., food), with the potential to put important financial pressure on HCW, is expected in the current crisis.

International collaboration in health human resources tailored to country-specific epidemiology (e.g., disease pattern) and cultural context (e.g., local languages) might help LIC with poor health workforce density mitigate the immediate potential negative impact of the pandemic on the quality use of EMV. However, to improve efficiency and sustainability major focus should be directed to the exchange of HCW who, in addition to providing clinical services (needed to satisfy immediate needs to mitigate the negative impacts of the pandemic on use of medicines), could also be involved in capacity building to help LMIC develop their ability to generate efficiently quality health workforce (to improve preparedness of LMIC to future crises and avoid the risk of perpetual dependence of LMIC to imported HCW).

Moreover, a recent multi-national study across SSA showed that the prevalence of high health literacy is low on the continent: 35.8% in both sexes, 34.1% in females, and 39.2% in males [116]. This means that patients are another important contributor to poor compliance with medicines across the continent, regardless of the quality of prescriptions. Therefore, substantial investment in health literacy and numeracy of populations and patients as well as in health-literate healthcare organization are also necessary for optimal outcomes.

Overall, the role of human resources is critical to ensure quality use of EMV. Even though most of the strategies discussed here are important to mitigate the immediate risks posed or escalated by COVID-19, to ensure better outcomes in quality use of EMV in the mid- and long-term, further sustainable and comprehensive strategies are needed, including careful staff recruitment, detailed training, authorization to provide autonomous care, reliable data systems, and fair, performance-based compensation [117]. (Table 2).

### The need for global research and policy framework to develop missing essential medicines

Due to the persistent problem of unreliable supply systems, among other considerations, AU member states acknowledged the need for a local production of drugs during a meeting held in Abuja in 2005. The Pharmaceutical Manufacturing Plan for Africa (PMPA) to help gather the necessary partnerships and resources was therefore established and endorsed by 54 AU member states in Accra in 2007 [118]. When faced with the potential of increased national demand in drugs due the capacity of COVID-19 to overwhelm health systems and the uncertainty regarding the epidemic dynamics of COVID-19, some key producers of pharmaceutical products responded by halting the production of some medicines (deemed not a priority internally) and/or by restricting the export of certain medicines (to satisfy internal demands/risks). This further illustrates the need for African countries to develop their own capacity for pharmaceutical manufacturing (PM).

However, despite the adoption of PMPA over a decade ago, little progress has been accomplished in PM to date in Africa. As a result, < 2% of the medicines consumed across the continent are produced on the continent, with the rest being imported largely from India and China [76]. Only a few AU member states—Egypt, Morocco, South Africa, and Tunisia—have a sizeable capability for PM, but mainly for national consumption. Ghana, Kenya, Nigeria, and Tanzania are in the process of developing their PM capability. This little progress can be explained because production of medicines requires high-level human, technological, and financial capability. Low investment in research and development (R&D), poor medicines regulatory capacity, and other factors also pose critical challenges [49]. Most countries allocate < 1% of national health expenditure to R&D across the continent [74]. Restrictions posed by patents might also contribute, although this is less likely regarding EMV for most diseases that are MCDS on the continent. This is because most of these are off patent and could be produced in some AU member states (e.g., Egypt, Morocco, South Africa, and Tunisia) without the need for a high return-on-investment provided that the buyers (i.e., AU member states) can combine their efforts to pool their purchasing power. However, intellectual property rights (IPR) may still pose barriers to research, development, manufacturing, and supply of MP essential to combat COVID-19 and other severe pandemics.

Measures aimed at facilitating cross-border trade (e.g., the African Continental Free Trade Agreement—AfCFTA) and strategic cooperation in the region along with international technology transfer and sustainable financing are pivotal to help develop local PM capability on the continent [119, 120]. This is critical to improve the preparedness and resilience of AU member states to current and emerging vulnerabilities of the GSC. However, as discussed elsewhere [37], additional efforts are needed to perfect AfCFTA to ensure that the largest economies among AU member states (i.e., Nigeria, South Africa, and Egypt) do not dominate alone the markets across the continent. In relation to MP essential to combat pandemics such as COVID-19, waivers from certain provisions of IPR might be necessary to improve local PM capability [121]. Moreover, even though the geographical focus of the current analysis is Africa, we argue that improving the capability of AU member states to produce drugs is critical not only for Africa but also for global health security. This is because if more AU member states develop the capability to produce drugs, then the geographical diversity in PM could be increased. This would reduce global vulnerabilities arising from the dependence of many countries to (imported) drugs that are produced largely in a few countries. The lessons from the current crisis might provide the rationale as to why the IC and BP should support more AU member states in their efforts to develop local capability for PM to ensure global health security. Sustainable funding schemes by the IC and BP—grants instead of loans—are needed [37]. (Table 2).

### Limitations

Despite its utility for policymaking by providing a detailed account of what the prospective pathways and impacts of the COVID-19 pandemic on equitable access to EMV are likely to be across Africa, our analysis has important limitations. A major limitation is the speculative nature of the review. This is because the study is not a direct assessment of observed causal impact. Rather, the prospective pathways and impacts discussed are inferred based on epidemiological associations identified in previous studies and do not imply causality. Additionally, due to large heterogeneity across studies in outcome measures and sparsity of relevant quantitative data, we did not conduct meta-analysis to quantify probabilistically the uncertainty associated with each prospective pathway and impact. Therefore, empirical assessment at national and subnational levels, using field data, of the risks identified here should be a priority of future research. Our analytical strategy allows a comprehensive assessment of the risks that the COVID-19 pandemic poses to ASQA of EMV for UHC. Nevertheless, a more integrative approach could have also been considered in arriving at the study’s recommendations—which, having been tailored for specific dimensions, might result in some inconsistencies. Therefore, future studies should explore possible improvements in the analytical framework.

## Conclusions

Our study, the first of its scope, is of unique policy relevance across the continent. In addition to generating hypotheses for future studies, the risks discussed here and the associated policy recommendations may help vulnerable countries refine their response to the COVID-19 pandemic to ensure better outcomes. While current research has generally been focusing on drugs relevant for COVID-19, in this study, we showed that, for Africa in particular, it is the disruptive nature of the pandemic on the ASQA of EMV critical to control other MCDS across the continent that has the potential to create catastrophic consequences. Informed by current data, we argue that if the risks discussed in our analysis are not addressed carefully, the consequences might be greater than the direct burden of a counterfactual unmitigated COVID-19 on the continent.

CRA is vital to ensure that the limited resources are strategically gathered and allocated in view of other MCDS across Africa. In the face of the current disruption of the GSC, and with international partners increasingly focusing entirely on COVID-19 and internally, community-based innovative and sustainable strategies for SD are increasingly needed in Africa to ensure that local economic, social, demographic, and epidemiological risks and potentials are appropriately accounted for in COVID-19 responses across the continent. Strategic regional cooperation is needed to mitigate the effects of global and national vulnerabilities and improve the preparedness of African countries to emerging risks. If the current risks are addressed using integrated and sustainable solutions, the COVID-19 pandemic might become a catalyst for UHC: helping to ensure equity in access.

## Supplementary Information

**Additional file 1.**

## Data Availability

The data used in this analysis are drawn from the references provided.

## Abbreviations

$: United States Dollar
AfCFTA: African Continental Free Trade Agreement
AFTCOR: African Task Force for Coronavirus Preparedness and Response
ART: Antiretroviral therapy
ARV: Antiretroviral drugs
ASQA: Accessibility, safety, quality, and affordability
AU: African Union
BP: Bilateral partners
CDC: Centres for Disease Control and Prevention
CHW: Community health worker
COVID-19: Coronavirus disease 2019
CRA: Comparative risk assessment
DRC: Democratic Republic of the Congo
EMV: Essential medicines and vaccines
GDP: Gross domestic product
GNI: Gross national income
GSC: Global supply chain
HCW: Healthcare workers
HIC: high-income countries
HIV/AIDS: Human immunodeficiency virus infection and acquired immune deficiency syndrome
IC: International community
ITN: Insecticide-treated bed nets
IPR: Intellectual property rights
LIC: Low-income countries
LMIC: Low- and middle-income countries
LY: Life-years
MC: Mobile clinics
MCDS: Major causes of death and suffering
MMR: Measles, mumps, and rubella
MMRV: Measles, mumps, rubella, and varicella
MP: Medical products
ODA: Official development assistance
PEPFAR: President’s Emergency Plan for AIDS Relief
PM: Pharmaceutical manufacturing
PLWHA: People living with HIV/AIDS
PRISMA: Preferred Reporting Items for Systematic Reviews and Meta-Analyses
QALYs: Quality-adjusted life-years
R&D: Research and development
SARS-CoV-2: Severe acute respiratory syndrome coronavirus 2
SD: Service delivery
SSA: Sub-Saharan Africa
UHC: Universal health coverage
WHO: World Health Organization
