# Supplementary material for: A review of prospective pathways and impacts of COVID-19 on the accessibility, safety, quality, and affordability of essential medicines and vaccines for universal health coverage in Africa
Source: Global Health. 2021 Apr 8;17:42. doi: 10.1186/s12992-021-00666-8 (PMC8027968; doi:10.1186/s12992-021-00666-8)
Supplement: Supplementary file 1 — Additional file 1. [file 12992_2021_666_MOESM1_ESM.docx]

Supplementary appendix:

Supplement to: A review of prospective pathways and impacts of COVID-19 on the accessibility, safety, quality, and affordability of essential medicines and vaccines for universal health coverage in Africa

Table of contents:

[**Supplement 1: Extended results** 2](#_Toc53323191)

[**Supplement 2: Further references** 11](#_Toc53323192)

# **Supplement 1:**

Extended results

**Table S1: Outline of eligible studies across applicable modules and domains. Module 1=evidence relevant for the assessment of the potential/observed pathways and impacts of COVID-19 on service delivery and/or equitable access to quality medicines and vaccines. Module 2=evidence on innovations in service delivery and/or management and supply of essential medicines and vaccines applicable to manage the risks potentially associated with the COVID-19 pandemic. Domain 1=assuring the financing and supply of essential medicines and vaccines. Domain 2=making essential medicines and vaccines affordable. Domain 3=assuring the quality and safety of medicines to prevent harm to patients. Domain 4=promoting quality use of essential medicines to ensure better health outcomes. Domain 5=the need for global research and policy framework to develop missing essential medicines. ACT=artemisinin-based combination therapy. AL=artemether‐lumefantrine. ART=antiretroviral therapy. AYA=adolescents and young adults. BRICS=Brazil, Russia, India, China, and South Africa. CD4=cluster of differentiation 4. CHW=community health workers. CNPs=clinical nurse practitioners. COVID-19=coronavirus disease 2019. CDs=communicable diseases. CRD=chronic rheumatic diseases. CSCS=COVID-19 Supply Chain System. DALYs=disability-adjusted life years. DOTS=directly observed treatment, short-course. DRC=Democratic Republic of the Congo. EAC=East African Community. EmOC=emergency obstetric care. EM=essential medicines. EVD=Ebola virus disease. FBC=facility-based care. FBO=faith-based organisations. FCAS=fragile and conflict-affected settings. FDCP=free delivery and caesarean policy. FP=family planning. GPs=general practitioners. HBC=home-based care. HB-HTC=home-based HIV testing and counselling. HC=health centres. HCW=healthcare workers. HF=healthcare facility. HIV/AIDS=human immunodeficiency virus infection and acquired immune deficiency syndrome. HL=health literacy. HPB=hepato-pancreato-biliary. IEC=information, education, and communication. IPR=intellectual property rights. IPTp=**i**ntermittent preventive treatment in pregnancy. IT=intervention theory. KSA=Kingdom of Saudi Arabia. KTHC=Klerksdorp-Tshepong Hospital Complex. KZN=KwaZulu-Natal. LMIC=low- and middle-income countries. MC=mobile clinic. MCC=mobile clinic care. MCH=maternal health. MC-HTC=mobile clinic HIV testing and counselling. MENA=Middle East North Africa.** **MRH=medicines regulatory harmonization. MRT=middle-range theories. MSM=men who have sex with men. NA=not available. NCDs=non-communicable diseases. NPHWs=nonphysician health workers. OOP=out-of-pocket. PBF=performance-based financing. PEPFAR=President's Emergency Plan for AIDS Relief. PHC=primary health care. PM=pharmaceutical manufacturing. POC=point-of-care. PrEP=pre-exposure prophylaxis. RTS=return to sport. SA=South Africa. SARS-CoV-2=severe acute respiratory syndrome coronavirus 2. SD=service delivery. SEA=southeast Asia.** **SRHS=sexual and reproductive health service. SSA=sub-Saharan Africa. TBAs=traditional birth attendants. TPT=tuberculosis preventive therapy. TRIPS=Trade-Related Aspects of Intellectual Property Rights. UAE=United Arab Emirates. UFEPs=user fee exemption policies. US=United States. WHO=World Health Organization.**

| # | Study | Scope | Regions | Domains | Modules |
| --- | --- | --- | --- | --- | --- |
| 1 | Abbas et al, 2020^1^ | The study estimated the benefit–risk ratio by comparing the deaths prevented by sustaining routine childhood immunisation for diphtheria, tetanus, pertussis, hepatitis B, Haemophilus influenzae type b, Streptococcus pneumoniae, rotavirus, measles, Neisseria meningitidis serogroup A, rubella, and yellow fever with the excess COVID-19 deaths associated with vaccination clinic visits. | Africa | 2 | 1 |
| 2 | African Union, 2007^2^ | The report describes pharmaceutical manufacturing plan for Africa | Africa | 5 | 2 |
| 3 | Ahmed et al, 2020^3^ | The study assessed stakeholder perspectives on access and barriers to healthcare among Slum residents’ over two time periods: pre-COVID-19 and during COVID-19 lockdowns. | Slum communities of Bangladesh, Kenya, Nigeria and Pakistan | 2 | 1 |
| 4 | Akoria et al, 2008^4^ | The study investigated (1) how far prescriptions meet accepted standards, (2) factors underlying poor prescription writing, and (3) the effects of intervention | Benin City, Southern Nigeria | 4 | 1,2 |
| 5 | Al-Ali et al, 2020^5^ | The study (1) addressed the gendered implications of Covid-19 in the Global South by paying attention to the intersectional pre-existing inequalities that have given rise to specific risks and vulnerabilities, (2) explored various aspects of the pandemic-induced ‘crisis of social reproduction’ that affects women as the main caregivers, and (3) examined the drastic increase of various forms of gender-based violence | Global South | 2 | 1 |
| 6 | Amazigo et al, 1998^6^ | The study examined effective system of ivermectin (Mectizan) delivery, involving drug procurement, delivery from port to districts and distribution to eligible persons | Onchocerciasis-endemic countries | 2 | 2 |
| 7 | Amimo et al, 2020^7^ | The study analysed potential pathways by which the COVID-19 responses across Africa might result in reduced capacity of patients to use essential health services | Africa | 2,3 | 1 |
| 8 | Amin et al, 2007^8^ | The study reviewed literature on sub-standard and counterfeit antimalarial drugs circulating in the markets of the African continent | Africa | 3 | 1 |
| 9 | Aneni E et al 2013^9^ | The study assessed the health changes of orphans and other vulnerable and non-vulnerable children visiting a MC in rural Namibia | Rural Namibia | 2 | 2 |
| 10 | Angwenyi et al, 2020^10^ | The study investigated current practices of delivering and implementing integrated care for chronically ill patients in rural Malawi, focusing on the primary level | Rural Malawi | 2 | 2 |
| 11 | Anyakora et al, 2018^11^ | The study assessed the quality of oxytocin injection, misoprostol tablets, magnesium sulphate, and calcium gluconate injections | 6 geopolitical zones of Nigeria: Abia State for the southeast, Edo State for the south-south, Ogun tate for the southwest, Kwara State for the north central, Kaduna State for the northwest, and Bauchi State for the northeast. | 3 | 1 |
| 12 | Arndt et al, 2020^12^ | The study examined the implications of lockdown policies for income distribution and food security, using SA as a case country | SA | 2,4 | 1 |
| 13 | Ashengo et al, 2014^13^ | The study investigated (1) the reasons voluntary medical male circumcision (VMMC) clients called the VMMC hotline and (2) the accuracy of telephone-based triage for VMMC in differentiating among mild, moderate, and severe adverse events (AEs) | Eswatini | 4 | 2 |
| 14 | Babigumira et al, 2009^14^ | The study compared the cost effectiveness of HBC, FBC and MCC for provision of ART in Uganda | Uganda | 2,4 | 2 |
| 15 | Bagayoko et al, 2011^15^ | The study described factors that might compromise the applicability of ICT as a powerful vector of balance in health systems but also a way to promote equal access to care in SSA countries | Mali | 2 | 2 |
| 16 | Bailie R, 1996^16^ | The study analysed the cost and cost-effectiveness of cervical cytology screening via a MC compared with that of screening at established clinics | SA | 2 | 2 |
| 17 | Bains et al, 2011^17^ | The study assessed associations among health literacy, diabetes knowledge, self-care, and glycaemic control in 18+ years of age, low income, minority population, with type 2 diabetes mellitus | Charleston, South Carolina, US | 4 | 1 |
| 18 | Balakrishnan et al, 2020^18^ | The study surveyed the impact of COVID-19 on HPB surgery | Africa, Europe | 2 | 1 |
| 19 | Balvanz et al, 2019^19^ | The study assessed individual and contextual factors associated with successful loan repayment among young men participating in a microfinance and health intervention in Dar es Salaam, Tanzania | Dar es Salaam, Tanzania | 2 | 2 |
| 20 | Bate et al, 2008^20^ | The study sampled the quality of medicines throughout the geographic band of hyper- and holoendemic *P. falciparum* malaria | Ghana, Kenya, Nigeria, Rwanda, Tanzania, Uganda | 3 | 1 |
| 21 | Belew et al, 2018^21^ | The study assessed pharmaceutical quality of albendazole, mebendazole and praziquantel tablets brands commonly available in legally operating retail pharmacies | Jimma, Ethiopia | 3 | 1 |
| 22 | Bell et al, 2020^22^ | The study compared theoretical mortality and (DALYs lost with the impact of possible scenarios of the COVID-19 public health response on morbidity and mortality for HIV/AIDS, malaria, and MH in Uganda | Uganda | 2 | 1 |
| 23 | Boyer et al, 2010^23^ | The study assessed the impact of decentralization of SD, comparing three levels of ART delivery (centralized, provincial, district-level) in terms of patients’ outcomes and accessibility of HC | Cameroon | 2 | 1,2 |
| 24 | Brenton et al, 2012^24^ | The study analyses the potential benefits and associated issues of regional trade integration in goods and services in Africa | Africa | 5 | 2 |
| 25 | Brey et al, 2020^25^ | The study examined the functioning and associated risks and potentials of home delivery of medication in patients with chronic conditions instituted by the Metropolitan Health Services (MHS) to reduce the risk of COVID-19 | Cape Town, SA | 2 | 2 |
| 26 | Buonsenso et al, 2020^26^ | The study investigated the potential indirect impact of COVID-19 on child vaccinations and basic healthcare in a typical poor peripheral area of Sierra Leone in children under 5 years of age | Kent, Rural Western Area, Sierra Leone | 2 | 1 |
| 27 | Cabore et al, 2020^27^ | The study (1) modelled the potential effects of widespread community transmission of SARS-CoV-2 infection in the WHO African region, and (2) examined potential detrimental impacts of COVID-19 on health system capacity | WHO African region | 2 | 1 |
| 28 | Carbaugh et al, 2020^28^ | The report analyses interruptions, delays, and other challenges in the HIV service ecosystem that are likely to occur due to the COVID-19 pandemic and summarizes PEPFAR’s response to COVID-19 in developing countries. | PEPFAR countries | 1,2,4 | 1,2 |
| 29 | Chu et al, 2020^29^ | The study (1) described changes in SA hospital surgical practices in response to COVID­-19 prepar­edness during the national lockdown, and (2) examined the potential consequences of reduced access to surgical care during lockdown | Africa | 2 | 1 |
| 30 | Cilloni et al, 2020^30^ | The study modelled the potential TB-related impact of COVID-related lockdowns - and mitigating effects of potential post-lockdown interventions - in three focal countries | India, the Republic of Kenya, and Ukraine | 2 | 1 |
| 31 | Coeytaux et al, 1987^31^ | The study compared the effectiveness to increases the use of FP services between (1) supplementing existing services with MC based implementation of an IEC outreach program, and (2) augmenting the number of service sites | Rural region of Tunisia: governorate of Mahdia | 2 | 2 |
| 32 | Coker et al, 2020^32^ | The study reviewed the potential impacts of COVID-19 on the prevention and control of other CDs and NCDs in children aged 0-19 years | SSA | 2 | 1 |
| 33 | Dansie et al, 2019^33^ | The study assessed pharmaceutical companies’ perceptions and experiences with the EAC MRH initiative, especially how well it encourages them to serve the EAC market | Eastern Africa | 3 | 1,2 |
| 34 | Davey et al, 2020^34^ | The study assessed the effect of national COVID-19 lockdown on study visits and PrEP prescriptions to HIV-negative pregnant women in antenatal care in SA | SA | 2 | 1 |
| 35 | De Beer et al, 2015^35^ | The study (1) assessed the costs of mobile voluntary counselling and testing (VCT) and wellness service delivery and (2) compared the costs and effectiveness with fixed VCT | Namibia | 2 | 2 |
| 36 | Dionisio et al, 2006^36^ | The study (1) explored key issues, implications, and interaction dynamics across in relation to the risks that enforced IPR pose to equitable HIV treatment access by poor populations, and (2) provided equitable solution glimpses | Under-Served Markets | 2,5 | 1,2 |
| 37 | Dorward et al, 2017^37^ | The study examined (1) verified linkage-to-care by 365 days after testing HIV-positive from a mobile HIV counselling and testing (HCT), (2) factors associated with changing referral clinic (defined as verified linkage-to-care at a different clinic from the clinic they chose or were referred to at enrolment) | Gauteng and Limpopo provinces, SA | 2 | 2 |
| 38 | Douglas et al, 2020^38^ | The analysis examines the mechanisms and mitigation measures of likely effects of social distancing measures to control the spread of covid-19 on low-income populations | Global | 2 | 1,2 |
| 39 | Ekeigwe et al, 2019^39^ | The study (1) investigated the problems of drug manufacturing and challenges of accessing medicines in West Africa, and (2) evaluated the strategies being adopted by international developmental partners and the governments to address them | West Africa | 1-5 | 1 |
| 40 | Essack et al, 2011^40^ | The study (1) examined the regulatory environment and supply chain for antibiotics for both the public and private sectors, and (2) reviewed what is known about patterns of antibiotic consumption in SA | SA | 1-3 | 1 |
| 41 | Ezziane et al, 2014^41^ | The study examined opportunities in drug production in inter-collaborations among BRICS, as wells as BRICS and developing countries | BRICS | 5 | 2 |
| 42 | Fiehler et al, 2020^42^ | The study surveyed the provision of neurointerventional services under COVID-19 conditions worldwide | Global | 2 | 1 |
| 43 | Fox-Rushby JA, 1995^43^ | The study evaluated the cost-effectiveness of MC to overcome problems of access to maternal health services among rural populations | Rural districts of the Gambia: West Kiang (MC), Upper Baddibu (comparator) | 2 | 2 |
| 44 | Frehywot et al, 2013^44^ | The study (1) reviewed the literature on e-learning for improved access to medical education, and (2) proposed framework for the implementation | LMIC | 4 | 1,2 |
| 45 | Freudenberg et al, 2020^45^ | The study evaluated the impact of COVID-19 on nuclear medicine departments | Global | 1,2 | 1 |
| 46 | Gavi, the Vaccine Alliance, 2020^46^ | The report describes activities that Gavi has taken to support the financing and supply of medical products relevant for the fight against the COVID-19 pandemic in LIC | LIC | 2 | 1,2 |
| 47 | Geldsetzer et al, 2020^47^ | The study measured the travel time to the nearest HF of any type for populations aged +60 years in SSA | SSA | 2 | 1 |
| 48 | Gnegel et al, 2020^48^ | The study assessed the quality of chloroquine tablets to illustrate the risks of falsified COVID-19 products entering LMICs markets | Cameroon and the Democratic Republic of Congo | 3 | 1 |
| 49 | Gorman et al, 2015^49^ | The study investigated whether the introduction of semi-mobile clinics had a positive impact on HIV infection outcomes compared with those who continued to use the hospital | Rural Kenya: West Pokot County | 2 | 2 |
| 50 | Govindasamy et al, 2013^50^ | The study examined the yield of newly diagnosed HIV, tuberculosis symptoms, diabetes, and hypertension, and to assess CD4 count testing, linkage to care as well as correlates of linkage and barriers to care from a mobile testing unit | Cape Town, SA | 2 | 2 |
| 51 | Harries et al, 2007^51^ | The study described an ART procurement model that allows rational drug forecasting and uninterrupted drug supplies and the lessons learned | Malawi | 1 | 2 |
| 52 | Heller et al, 2019^52^ | The study reviewed the literature on barriers to and facilitators of NPHWs led care for NCDs in LMIC | LMIC | 4 | 1,2 |
| 53 | Hogan et al, 2020^53^ | The study predicts potential impacts on HIV, TB, malaria burden under hypothetical scenarios of health service disruptions due to the COVID-19 pandemic | LMIC | 2 | 1 |
| 54 | Hsiao et al, 2019^54^ | The study evaluated the relationship between paying bribes and reported difficulties of obtaining medical care in adult patients | SSA | 2,3 | 1 |
| 55 | Ibrahim et al, 2020^55^ | The study reviewed the opportunities and challenges faced by researchers in the MENA region to conduct high quality clinical trials during the Covid-19 pandemic | MENA | 5 | 2 |
| 56 | Igumbor et al, 2016^56^ | The study assessed the amount of time that CNPs in one district of the Western Cape spend on clinical services, comparing fixed with mobile clinics | District of the Western Cape, SA | 2 | 2 |
| 57 | International Food Policy Research Institute, 2020^57^ | The analysis projected likely impacts of the COVID-91 related economic downturn on poverty worldwide and regionally | Global | 1,2 | 1 |
| 58 | International Monetary Fund, 2020^58^ | The analysis projects economic implications of the COVID-19 pandemic | Global | 1,2 | 1 |
| 59 | Jacobs et al, 2020^59^ | The study examined the role of PBF with reference to: (1) user fees/exemption policies and (2) basic packages of health services and benefit packages | FCAS: Central African Republic, Democratic Republic of the Congo, and Nigeria | 1,2 | 2 |
| 60 | Jacobson JL, 1991^60^ | The study described the feasibility of, and risks associated with, the implementation of TBAs as a national public health strategy in Zimbabwe | Zimbabwe | 2 | 1,2 |
| 61 | Jewell et al, 2020^61^ | The study modelled the potential effects of HIV service disruptions in SSA resulting from COVID-19 | SSA | 2 | 1 |
| 62 | Jewell et al, 2020^62^ | The study predicts potential impacts on HIV burden under hypothetical scenarios of health service disruptions due to the COVID-19 pandemic | SSA | 2 | 1 |
| 63 | Joint United Nations Programme on HIV and AIDS, 2020^63^ | The report analyses the impacts that the COVID-19 pandemic could have on HIV burden and control in LMIC | Global | 2,5 | 1 |
| 64 | Kay et al, 2016^64^ | The study explored the barriers to and facilitators of quality use of medicines experienced by refugees, pharmacists, GPs, and nurses at a PHC setting | Australia | 4 | 1,2 |
| 65 | Kefale et al, 2019^65^ | The study assessed EM availability and inventory management practices at HC of Adama town, Ethiopia | Adama town, Ethiopia | 1 | 1,2 |
| 66 | Kranzer et al, 2012^66^ | The study investigated the feasibility, uptake, treatment outcomes, and cost of adding an active TB case-finding program linked to an existing mobile HIV testing service in Cape Town, SA | Cape Town, SA | 2 | 2 |
| 67 | Kredo et al, 2014^67^ | The study reviewed the quality of initiation and maintenance of HIV/AIDS care in models that task shift care from doctors to non-doctors | Africa | 4 | 2 |
| 68 | Kroon et al, 2001^68^ | The study analysed the data for treatment performed in the dental clinic from using MC | SA | 2 | 2 |
| 69 | Labhardt et al, 2014^69^ | The study compared HB-HTC with MC-HTC to test which programmatic approach would be more effective for a rural, high-prevalence setting | Rural Lesotho (district of ButhaButhe in northern Lesotho and district of Thaba-Tseka in central Lesotho) | 2 | 2 |
| 70 | Larson et al, 2012^70^ | The study assessed the proportion of patients who completed their referral visit within 8 weeks of HIV testing in the mobile program under routine conditions and evaluate the impact of including POC CD4 testing on the proportion of patients completing the referral visit within 8 weeks of HIV testing | SA | 2 | 2 |
| 71 | Lewis et al, 2009^71^ | The study examined the role of good governance to raising performance in health care delivery | Global | 2,3 | 2 |
| 72 | Libamba E et al, 2005^72^ | The study explored the feasibility of using DOTS to deliver ART to large numbers of HIV-infected patients under controlled conditions, and minimize the risk of developing drug resistance | Malawi | 3,4 | 2 |
| 73 | Masiye et al, 2010^73^ | The study evaluated the performance of user fees abolition policy at primary health care facilities in designated rural districts in Zambia, following 15 months of implementation | Designated rural districts in Zambia | 2 | 2 |
| 74 | Mavungu et al, 2019^74^ | The study evaluated the existing pharmaceutical system in peri-urban areas of Kinshasa, mainly with regard to the use of antimalarials and their quality, the proportion of unlicensed antimalarials and the characteristics of pharmaceutical establishments | Kinshasa peri-urban areas, Democratic Republic of Congo | 3 | 1 |
| 75 | Mc Donald T, Chapman RD and MacKenzie J, 1994^75^ | The study assessed aspects of the nurses’ work in MC that can be computerized to improve quality of work | Orange Free State, SA | 2,4 | 2 |
| 76 | McClintock et al, 2019^76^ | The study (1) developed and evaluated a HL measure in a multi-national study and (2) examined demographic characteristics associated with HL, in populations aged 15 and 49 years | SSA | 4 | 1 |
| 77 | McQuaid et al, 2020^77^ | The study estimated the relative impact of reductions in social contacts and health services due to COVID-19 on TB burden | China, India, and SA | 2 | 1 |
| 78 | Mhimbira et al, 2017^78^ | The study reviewed the effectiveness of different strategies to increase tuberculosis case detection through improving access (geographical, financial, educational) to tuberculosis diagnosis at PHC or community-level services | Global: SSA (Ethiopia, Nigeria, SA, Zambia, and Zimbabwe), Asia (Bangladesh, Cambodia, India, Nepal, and Pakistan), and South America (Brazil and Colombia) | 2 | 2 |
| 79 | Micah et al, 2019^79^ | The study assessed the growth in government health spending, examine its determinants and explain the variation in government health spending across SSA countries | SSA | 1,5 | 1 |
| 80 | Miyano et al, 2017^80^ | The study analysed the cost effectiveness of the National Mobile ART Services Programme in Zambia as a means of decentralizing ART services | Zambia | 2 | 2 |
| 81 | Moore et al, 2020^81^ | The report describes the applicability of telemedicine to improve access to healthcare and reduce HCW workload in the context of the COVID-19 pandemic | SA | 2, 4 | 2 |
| 82 | Moustakis et al, 2020^82^ | The study examined the change in urgent and emergency admissions to the Department of Surgery at KTHC prior to and during the first stage of COVID-19 lockdown | North West, SA | 2 | 1 |
| 83 | Mubyazi et al, 2008^83^ | The study reviewed literature on policy advances, achievements, constraints, and challenges to malaria IPTp implementation, with focus on its operational feasibility in the context of health-care financing, provision and uptake, resource constraints and psychosocial factors in Africa | Africa | 2 | 1 |
| 84 | Mufusama et al, 2018^84^ | The study assessed the quality of antimalarial medicines: AL | 8 main cities in the DRC: Goma, Kikwit, Kinshasa, Kisangani, Lubumbashi, Matadi, Mbandaka, and Mbuji‐Mayi | 3 | 1,2 |
| 85 | Mwambete et al, 2009^85^ | The study assessed the prevalence of antibiotic misuse among boarding secondary school students aged 19-21 years in Dar es Salaam | Dar es Salaam, Tanzania | 4 | 1 |
| 86 | NA, 1979^86^ | The study assessed feasibility of moving from the traditional clinic-based FP program to a door-to-door contraceptive distribution system | Rural Tunisia | 2 | 2 |
| 87 | Nayyar et al, 2012^87^ | The study reviewed published and unpublished studies reporting chemical analyses and assessments of packaging of antimalarial drugs | SEA and SSA | 3 | 1,2 |
| 88 | Nguyen et al, 2020^88^ | The study estimated the cost-effectiveness of 2 user-fee exemption interventions in Burkina Faso between 2007 and 2015: the national 80% user-fee reduction policy for delivery care services and the user-fee removal pilot (i.e., the complete [100%] user-fee removal for delivery care) in the Sahel region | Burkina Faso | 2 | 2 |
| 89 | Nyato et al, 2018^89^ | The study reviewed evidence on accrual, delivery strategies and uptake of services among MSM in SSA | SSA | 2 | 2 |
| 90 | O’Doherty et al, 2018^90^ | The study reviewed the literature on known barriers and solutions that face educators when developing and implementing online learning programs for medical students and postgraduate trainees | Global | 4 | 1,2 |
| 91 | Onakpoya et al, 2015^91^ | The study investigated medical products that were withdrawn after marketing in association with deaths | Global | 3 | 1,2 |
| 92 | Onwujekwe et al, 2019^92^ | The study reviewed literature on corruption involving front-line healthcare providers, their managers, and other stakeholders in health sectors | 5 Anglophone West African countries: Gambia, Ghana, Liberia, Nigeria and Sierra Leone | 2,3 | 1,2 |
| 93 | Organisation for Economic Co-operation and Development, 2018^93^ | The analysis (1) identifies factors that make health systems unable to ensure that innovative medicines are affordable and (2) provides policy options for policy makers to consider in order to improve the current system | Global | 1,2,3 | 1,2 |
| 94 | Oualla et al, 2020^94^ | The paper described the changes in SD in anticancer centres in response to COVID-19 ensure the continuation of adequate cancer care | Morocco | 2 | 1,2 |
| 95 | Palmer et al, 2016^95^ | The study described the strategies used by eye care practitioners in four hospitals of north-west Tanzania to navigate the government, mission, FBO and donor rules that govern eye services delivery in the country to ensure financial sustainability | North-west Tanzania | 2 | 2 |
| 96 | Parmar et al, 2019^96^ | The study examined the equity implications of two interventions on both supply- and demand-sides of health system in the context of maternal healthcare in Senegal: delegation of EmOC to non-physicians (supply-side intervention) and FDCP in public health facilities (demand-side intervention) | Senegal | 2 | 2 |
| 97 | Pathmanathan et al, 2018^97^ | The report examined barriers and applicable solutions to scaling-up TPT in resource-limited settings | Resource-limited settings | 2,3,5 | 1,2 |
| 98 | Pheage et al, 2017^98^ | The article describes factors inhibiting pharmaceutical local production in Africa, suspicions of African political leaders in relation to reluctance of world’s biggest pharmaceutical companies to offer technical support to African manufacturers, progress in some countries in pharmaceutical manufacturing, and recourse to traditional medicines due to difficulties in accessing modern medicines | Africa | 5 | 1 |
| 99 | Pillay et al, 2020^99^ | The study examined (1) perceptions of SA elite and semi-elite athletes on RTS, (2) maintenance of physical conditioning and other activities, (3) sleep, (4) nutrition, (5) mental health, (6) healthcare access, and (7) knowledge of COVID-19 | SA | 2 | 1 |
| 100 | Ponsar et al, 2011^100^ | The study (1) examined the impact of user fees on healthcare-seeking behaviour and access, and (2) measured the impact of reduced payment systems in Chad, Mali, Haiti and Burundi, and user fee abolition for certain population groups in Burundi and Mali | Burundi, Sierra Leone, Democratic Republic of Congo, Chad, Haiti, and Mali | 2 | 1,2 |
| 101 | Ravit et al, 2018^101^ | The study examined whether there was an increase or a decrease in urban/rural and socioeconomic inequalities in access to C-sections and facility-based deliveries after the free C-section policy was introduced | Benin and Mali | 2 | 2 |
| 102 | Ravit et al, 2018^102^ | The study assessed the impact of user fee exemption policy on caesarean sections on service utilisation and neonatal outcomes | Western Africa: Mali and Benin | 2 | 2 |
| 103 | Ridde et al, 2015^103^ | The study assessed the equity impact on household OOP spending for facility-based delivery of the user fee reduction policy implemented in Burkina Faso since 2007 | Burkina | 2 | 2 |
| 104 | Robert et al, 2017^104^ | The study (1) presented the theoretical explanation of how UFEPs are supposed to produce their intended outcomes (the IT) and (2) produced MRT of free public healthcare seeking in SSA | SSA | 2 | 2 |
| 105 | Roberton et al, 2020^105^ | The study quantified the potential indirect effect of the COVID-19 pandemic and control efforts on reproductive, maternal, newborn, and child health in 118 LMIC using the Lives Saved Tool | LMIC | 2 | 1 |
| 106 | Roth et al, 2018^106^ | The study reviewed global normative documents and resources and engaged with global partners and stakeholders to identify critical challenges faced by national regulatory authorities limiting access to medical products and impeding detection of and response to substandard or falsified drugs | LMIC | 3 | 1,2 |
| 107 | Sagaon‐Teyssier et al, 2016^107^ | The study (1) analysed the transactional data on donor-funded ARV procurement and the ARV price determinants between 2003 and 2015, and (2) provided a landscape of the global ARV market | LMIC | 5 | 2 |
| 108 | Semo et al, 2020^108^ | The review examined potential mental health impact of the COVID-19 Pandemic in SSA | SSA | 2 | 1 |
| 109 | Sherrard-Smith et al, 2020^109^ | The study predicts potential impacts on malaria burden under hypothetical scenarios of health service disruptions due to the COVID-19 pandemic | Africa | 2 | 1 |
| 110 | Siedner et al, 2020^110^ | The study examined how the implementation of the nationwide lockdown (shelter-in-place) order in SA affected ambulatory clinic visitation in rural KZN | Rural KZN, SA | 2 | 1 |
| 111 | Smith et al, 2019^111^ | The study investigated patient acceptability of mobile AYA SRHS and compare mobile clinic usage and HIV outcomes with nearby conventional clinics | Cape Town, SA | 2 | 2 |
| 112 | Stadler et al, 2006^112^ | The study examined acceptability of a brothel-based clinic to female sex workers in terms of service quality, accessibility, and efficacy, and positively influenced health-seeking behaviours, health awareness, and condom use | Hillbrow, SA | 2 | 2 |
| 113 | Stormacq et al, 2020^113^ | The study reviewed evidence on the effectiveness of HL interventions in socioeconomically disadvantaged people living in the community for improving health-related outcomes | Global | 4 | 1,2 |
| 114 | Suleman et al, 2016^114^ | The study assessed the pharmaceutical regulatory system in Ethiopia and examined possible reasons for deficiencies in the pharmaceutical chain | Ethiopia | 3 | 1 |
| 115 | Surie et al, 2019^115^ | The study assessed current status of TPT implementation in countries supported by PEPFAR | PEPFAR-supported countries covered: Angola, Botswana, Cambodia, Cameroon, China, Côte d'Ivoire, Democratic Republic of the Congo, Dominican Republic, Eswatini, Ethiopia, Ghana, Guatemala, Haiti, India, Jamaica, Kenya, Kyrgyzstan, Laos, Lesotho, Malawi, Mali, Mozambique, Myanmar, Namibia, Nigeria, Papua New Guinea, Rwanda, SA, South Sudan, Tanzania, Thailand, Uganda, Viet Nam, Zambia, Zimbabwe | 1 | 1 |
| 116 | Taylor et al, 2017^116^ | The study assessed the affordability of rural CHW programmes by estimating total programme costs relative to national healthcare expenditure at different CHW salaries and resources available for healthcare | SSA | 2,4 | 2 |
| 117 | Teachout et al, 2020^117^ | The analysis projects economic implications of the COVID-19 pandemic | Global | 1,2 | 1 |
| 118 | The World Bank, 2020^118^ | The analysis projects economic implications of the COVID-19 pandemic | Global | 1,2 | 1 |
| 119 | Tivura et al, 2016^119^ | The study determined the quality of ACT available in predominantly rural communities in the middle part of Ghana | 8 neighbouring districts of central part of Ghana: Kintampo North, Kintampo South, Nkoranza North, Nkoranza South, Techiman North, Techiman South, Tain and Wenchi | 3 | 1 |
| 120 | Togun et al, 2020^120^ | The study reviewed issues of how COVID-19 would affect TB control programmes: prioritisation of services, availability of drugs, the effect on hard-to-reach and LI communities and the role of stigmatisation, how paediatric TB disease might be affected, the possibility of increased disease transmission or disease susceptibility, and the problems caused by likely co-morbidity | Africa, UK | 2 | 1 |
| 121 | United Nations Economic Commission for Africa, 2020^121^ | The analysis predicted economic losses due to the COVID-19 pandemic across Africa | Africa | 1,2 | 1 |
| 122 | United Nations Secretary-General's High-Level Panel on Access to Medicines, 2016^122^ | The report describes the challenges and benefits of strengthening capacity for local PM in Africa | Africa | 3,5 | 2 |
| 123 | Wadoum et al, 2017^123^ | The study assessed the implementation of MC to address barriers related to uptake of services among EVD survivors | Rural communities of the Bombali and Port Loko districts of northern Sierra Leone, West Africa | 2 | 2 |
| 124 | Walker et al, 1976^124^ | The study compared MC with fixed clinics with regard to the cost-efficiency and cost-effectiveness of the delivery of PHC | Botswana | 2 | 2 |
| 125 | Walker et al, 1977^125^ | The study compared the cost-effectiveness of MC (air/land) with fixed HF: per patient contact and per likely effective patient contact | Botswana | 2 | 2 |
| 126 | Watson et al, 2018^126^ | The study reviewed the scope, quality, and findings of economic evaluations of service delivery interventions in LMIC | LMIC | 2,4 | 2 |
| 127 | Weiss et al, 2020^127^ | The study estimated the possible additional malaria-attributable morbidity and mortality across malaria-endemic African countries in 2020 relative to a baseline in which intervention deployment is unhindered | Africa | 2 | 1 |
| 128 | White et al, 2015^128^ | The study assessed the quality of baseline HC and measured associations with diabetes outcome | Tennessee, US | 4 | 1 |
| 129 | Wilkinson D and Sach ME, 1997^129^ | The study (1) investigated the feasibility, accuracy, and cost of developing a system of on-site screening for anaemia in pregnancy in primary care, and (2) described its adoption by a mobile clinic team | Hlabisa health district, Kwazulu-Natal, SA | 2 | 2 |
| 130 | Wirtz et al, 2017^130^ | The study identified five areas that are crucial to EM policies | Global | 1-5 | 1,2 |
| 131 | World Health Organization, 2020 1^131^ | The report describes the aims, structure, and functioning of the CSCS | Global | 1,2 | 1 |
| 132 | World Health Organization, 2020 2^132^ | The study predicts potential impacts on malaria burden under hypothetical scenarios of health service disruptions due to the COVID-19 pandemic | Africa | 2 | 1 |
| 133 | World Trade Organization, 2020^133^ | The report (1) describes barriers that intellectual property rights pose to global sharing of technology and know-how in order that rapid responses for the handling of COVID-19 can be put in place on a real time basis, and (2) requests a waiver from the implementation, application, and enforcement of Sections 1, 4, 5, and 7 of Part II of the TRIPS Agreement in relation to prevention, containment, or treatment of COVID-19. | SA, India | 5 | 2 |
| 134 | Ziadé et al, 2020^134^ | The study evaluated the impact of COVID-19 on the access to rheumatology care for patients with CRD in the Arab countries | Arab countries: Levant (Iraq, Jordan, Lebanon, Palestine, Syria), Gulf (Bahrain,Kuwait, Oman, Qatar, KSA, Oman, UAE) and North Africa (Algeria, Egypt, Libya, Morocco, Sudan, Tunisia) | 2 | 1 |

# **Supplement 2:**

Further references

1. Abbas K, Procter SR, van Zandvoort K, et al. Routine childhood immunisation during the COVID-19 pandemic in Africa: a benefit–risk analysis of health benefits versus excess risk of SARS-CoV-2 infection. *Lancet Glob Health* 2020; **8**(10): e1264-e72.

2. African Union. Pharmaceutical manufacturing plan for Africa. In: Third session of the African Union conference of Ministers of Health: strengthening of health systems for equity and development in Africa. 2017 April 9-13. Johannesburg. Addis Ababa: African Union, 2007.

3. Ahmed SAKS, Ajisola M, Azeem K, et al. Impact of the societal response to COVID-19 on access to healthcare for non-COVID-19 health issues in slum communities of Bangladesh, Kenya, Nigeria and Pakistan: results of pre-COVID and COVID-19 lockdown stakeholder engagements. *BMJ Glob Health* 2020; **5**(8): e003042.

4. Akoria OA, Isah AO. Prescription writing in public and private hospitals in Benin City, Nigeria: the effects of an educational intervention. *Can J Clin Pharmacol* 2008; **15**(2): e295-305.

5. Al-Ali N. Covid-19 and feminism in the Global South: challenges, initiatives and dilemmas. *Eur J Womens Stud* 2020; **27**(4): 333.

6. Amazigo U, Noma M, Boatin BA, Etya'ale DE, Seketeli A, Dadzie KY. Delivery systems and cost recovery in Mectizan treatment for onchocerciasis. *Ann Trop Med Parasitol* 1998; **92 Suppl 1**: S23-31.

7. Amimo F, Lambert B, Magit A. What does the COVID-19 pandemic mean for HIV, tuberculosis, and malaria control? *Trop Med Health* 2020; **48**: 32.

8. Amin AA, Kokwaro G. Antimalarial drug quality in Africa. *J Clin Pharm Ther* 2007; **32**(5): 429-40.

9. Aneni E, De Beer IH, Hanson L, Rijnen B, Brenan AT, Feeley FG. Mobile primary healthcare services and health outcomes of children in rural Namibia. *Rural Remote Health* 2013; **13**(3): 2380.

10. Angwenyi V, Angwenyi V, Angwenyi V, et al. Context matters: a qualitative study of the practicalities and dilemmas of delivering integrated chronic care within primary and secondary care settings in a rural Malawian district. *BMC Fam Pract* 2020; **21**(1): 101.

11. Anyakora C, Oni Y, Ezedinachi U, et al. Quality medicines in maternal health: results of oxytocin, misoprostol, magnesium sulfate and calcium gluconate quality audits. *BMC Pregnancy Childbirth* 2018; **18**(1): 44.

12. Arndt C, Davies R, Gabriel S, et al. Covid-19 lockdowns, income distribution, and food security: an analysis for South Africa. *Glob Food Sec* 2020; **26**: 100410.

13. Ashengo TA, Grund J, Mhlanga M, et al. Feasibility and validity of telephone triage for adverse events during a voluntary medical male circumcision campaign in Swaziland. *BMC Public Health* 2014; **14**(1): 858.

14. Babigumira JB, Sethi AK, Smyth KA, Singer ME. Cost effectiveness of facility-based care, home-based care and mobile clinics for provision of antiretroviral therapy in Uganda. *Pharmacoeconomics* 2009; **27**(11): 963-73.

15. Bagayoko CO, Anne A, Fieschi M, Geissbuhler A. Can ICTs contribute to the efficiency and provide equitable access to the health care system in sub-Saharan Africa? The Mali experience. *Yearb Med Inform* 2011; **6**: 33-8.

16. Bailie R. An economic appraisal of a mobile cervical cytology screening service. *S Afr Med J* 1996; **86**(9 Suppl): 1179-84.

17. Bains SS, Egede LE. Associations between health literacy, diabetes knowledge, self-care behaviors, and glycemic control in a low income population with type 2 diabetes. *Diabetes Technol Ther* 2011; **13**(3): 335-41.

18. Balakrishnan A, Lesurtel M, Siriwardena AK, et al. Delivery of hepato-pancreato-biliary surgery during the COVID-19 pandemic: an European-African Hepato-Pancreato-Biliary Association (E-AHPBA) cross-sectional survey. *HPB (Oxford)* 2020; **22**(8): 1128-34.

19. Balvanz P, Yamanis TJ, Mulawa MI, et al. Microfinance and health interventions: factors influencing loan repayment success with young men in Dar es Salaam, Tanzania. *Glob Public Health* 2019; **14**(2): 254-70.

20. Bate R, Coticelli P, Tren R, Attaran A. Antimalarial drug quality in the most severely malarious parts of Africa - a six country study. *PLoS One* 2008; **3**(5): e2132.

21. Belew S, Suleman S, Wynendaele E, et al. Quality of anthelminthic medicines available in Jimma Ethiopia. *Acta Trop* 2018; **177**: 157-63.

22. Bell D, Hansen KS, Kiragga AN, Kambugu A, Kissa J, Mbonye AK. Predicting the impact of COVID-19 and the potential impact of the public health response on disease burden in Uganda. *Am J Trop Med Hyg* 2020; **103**(3): 1191-7.

23. Boyer S, Eboko F, Camara M, et al. Scaling up access to antiretroviral treatment for HIV infection: the impact of decentralization of healthcare delivery in Cameroon. *AIDS* 2010; **24 Suppl 1**: S5-15.

24. Brenton P, Isik G. De-fragmenting Africa: deepening regional trade integration in goods and services. Washington, DC: World Bank Publications; 2012.

25. Brey Z, Mash R, Goliath C, Roman D. Home delivery of medication during coronavirus disease 2019, Cape Town, South Africa: short report. *Afr J Prim Health Care Fam Med* 2020; **12**(1): 2449.

26. Buonsenso D, Cinicola B, Kallon MN, Iodice F. Child healthcare and immunizations in sub-Saharan Africa during the COVID-19 pandemic. *Front Pediatr* 2020; **8**: 517.

27. Cabore JW, Karamagi HC, Kipruto H, et al. The potential effects of widespread community transmission of SARS-CoV-2 infection in the World Health Organization African Region: a predictive model. *BMJ Glob Health* 2020; **5**(5): e002647.

28. Carbaugh A, Kates J, Oum S. COVID-19 & PEPFAR: implications for the future. San Francisco: KFF, 2020.

29. Chu KM, Smith M, Steyn E, Goldberg P, Bougard H, Buccimazza I. Changes in surgical practice in 85 South African hospitals during COVID-19 hard lockdown. *S Afr Med J* 2020; **110**(9): 916-9.

30. Cilloni L, Fu H, Vesga JF, et al. The potential impact of the COVID-19 pandemic on the tuberculosis epidemic a modelling analysis. *EClinicalMedicine* 2020; **28**: 100603.

31. Coeytaux FM, Kilani T, McEvoy M. The role of information, education, and communication in family planning service delivery in Tunisia. *Stud Fam Plann* 1987; **18**(4): 229-34.

32. Coker M, Folayan MO, Michelow IC, Oladokun RE, Torbunde N, Sam-Agudu NA. Things must not fall apart: the ripple effects of the COVID-19 pandemic on children in sub-Saharan Africa. *Pediatr Res* 2020. doi: 10.1038/s41390-020-01174-y [Epub ahead of print].

33. Dansie LS, Odoch WD, Årdal C. Industrial perceptions of medicines regulatory harmonization in the East African Community. *PLoS One* 2019; **14**(6): e0218617.

34. Davey DLJ, Bekker L-G, Mashele N, Gorbach P, Coates TJ, Myer L. PrEP retention and prescriptions for pregnant women during COVID-19 lockdown in South Africa. *Lancet HIV* 2020; **7**(11): e735.

35. de Beer I, Chani K, Feeley FG, Rinke de Wit TF, Sweeney-Bindels E, Mulongeni P. Assessing the costs of mobile voluntary counseling and testing at the work place versus facility based voluntary counseling and testing in Namibia. *Rural Remote Health* 2015; **15**(4): 3357.

36. Dionisio D, Cao YZ, Lu HZ, Kraisintu K, Messeri D. Affordable antiretroviral drugs for the under-served markets: how to expand equitable access against the backdrop of challenging scenarios? *Curr HIV Res* 2006; **4**(1): 3-20.

37. Dorward J, Mabuto T, Charalambous S, Fielding KL, Hoffmann CJ. Factors associated with poor linkage to HIV care in South Africa: secondary analysis of data from the Thol'impilo trial. *J Acquir Immune Defic Syndr* 2017; **76**(5): 453-60.

38. Douglas M, Katikireddi SV, Taulbut M, McKee M, McCartney G. Mitigating the wider health effects of covid-19 pandemic response. *BMJ* 2020; **369**: m1557.

39. Ekeigwe AA. Drug manufacturing and access to medicines: the West African story. A literature review of challenges and proposed remediation. *AAPS Open* 2019; **5**(1): 3.

40. Essack SY, Schellack N, Pople T, et al. Part III. Antibiotic supply chain and management in human health. *S Afr Med J* 2011; **101**(8): 562-6.

41. Ezziane Z. Essential drugs production in Brazil, Russia, India, China and South Africa (BRICS): opportunities and challenges. *Int J Health Policy Manag* 2014; **3**(7): 365-70.

42. Fiehler J, Brouwer P, Diaz C, et al. COVID-19 and neurointerventional service worldwide: a survey of the European Society of Minimally Invasive Neurological Therapy (ESMINT), the Society of NeuroInterventional Surgery (SNIS), the Sociedad Iberolatinoamericana de Neuroradiologia Diagnostica y Terapeutica (SILAN), the Society of Vascular and Interventional Neurology (SVIN), and the World Federation of Interventional and Therapeutic Neuroradiology (WFITN). *J Neurointerv Surg* 2020; **12**(8): 726-30.

43. Fox-Rushby JA. The Gambia: Cost and effectiveness of a mobile maternal health care service, West Kiang. *World Health Stat Q* 1995; **48**(1): 23-7.

44. Frehywot S, Vovides Y, Talib Z, et al. E-learning in medical education in resource constrained low- and middle-income countries. *Hum Resour Health* 2013; **11**(1): 4.

45. Freudenberg LS, Paez D, Giammarile F, et al. Global impact of COVID-19 on nuclear medicine departments: an international survey in April 2020. *J Nucl Med* 2020; **61**(9): 1278-83.

46. Gavi, the Vaccine Alliance. COVID-19: Gavi steps up response to pandemic. Geneva: Gavi, the Vaccine Alliance, 2020.

47. Geldsetzer P, Reinmuth M, Ouma PO, et al. Mapping physical access to health care for older adults in sub-Saharan Africa and implications for the COVID-19 response: a cross-sectional analysis. *Lancet Healthy Longev* 2020; **1**(1): e32-e42.

48. Gnegel G, Hauk C, Neci R, et al. Identification of falsified chloroquine tablets in Africa at the time of the COVID-19 pandemic. *Am J Trop Med Hyg* 2020; **103**(1): 73-6.

49. Gorman SE, Martinez JM, Olson J. An assessment of HIV treatment outcomes among utilizers of semi-mobile clinics in rural Kenya. *AIDS Care* 2015; **27**(5): 665-8.

50. Govindasamy D, Kranzer K, Van Schaik N, et al. Linkage to HIV, TB and non-communicable disease care from a mobile testing unit in Cape Town, South Africa. *PLoS One* 2013; **8**(11): e80017.

51. Harries AD, Schouten EJ, Makombe SD, et al. Ensuring uninterrupted supplies of antiretroviral drugs in resource-poor settings: an example from Malawi. *Bull World Health Organ* 2007; **85**(2): 152-5.

52. Heller DJ, Kumar A, Kishore SP, Horowitz CR, Joshi R, Vedanthan R. Assessment of barriers and facilitators to the delivery of care for noncommunicable diseases by nonphysician health workers in low- and middle-income countries: a systematic review and qualitative analysis. *JAMA Netw Open* 2019; **2**(12): e1916545.

53. Hogan AB, Jewell BL, Sherrard-Smith E, et al. Potential impact of the COVID-19 pandemic on HIV, tuberculosis, and malaria in low-income and middle-income countries: a modelling study. *Lancet Glob Health* 2020; **8**(9): e1132-e41.

54. Hsiao A, Vogt V, Quentin W. Effect of corruption on perceived difficulties in healthcare access in sub-Saharan Africa. *PloS one* 2019; **14**(8): e0220583.

55. Ibrahim H, Kamour AM, Harhara T, Gaba WH, Nair SC. Covid-19 pandemic research opportunity: is the Middle East & North Africa (MENA) missing out? *Contemp Clin Trials* 2020; **96**: 106106.

56. Igumbor J, Davids A, Nieuwoudt C, Lee J, Roomaney R. Assessment of activities performed by clinical nurse practitioners and implications for staffing and patient care at primary health care level in South Africa. *Curationis* 2016; **39**(1): 1-8.

57. International Food Policy Research Institute. Poverty and food insecurity could grow dramatically as COVID-19 spreads. Washington, DC: International Food Policy Research Institute, 2020.

58. International Monetary Fund. World economic outlook, April 2020: the great lockdown. Washington, DC: International Monetary Fund 2020.

59. Jacobs E, Bertone MP, Toonen J, Akwataghibe N, Witter S. Performance-based financing, basic packages of health services and user-fee exemption mechanisms: an analysis of health-financing policy integration in three fragile and conflict-affected settings. *Appl Health Econ Health Policy* 2020; **18**(6): 801-10.

60. Jacobson JL. Maternal mortality and morbidity. Zimbabwe's birth force. *Newsl Womens Glob Netw Reprod Rights* 1991; (36): 16-7.

61. Jewell BL, Mudimu E, Stover J, et al. Potential effects of disruption to HIV programmes in sub-Saharan Africa caused by COVID-19: results from multiple mathematical models. *The lancet HIV* 2020; **7**(9): e629-e40.

62. Jewell BL, Smith JA, Hallett TB. Understanding the impact of interruptions to HIV services during the COVID-19 pandemic: a modelling study. *EClinicalMedicine* 2020; **26**: 100483.

63. Joint United Nations Programme on HIV and AIDS. Global HIV statistics. Geneva: Joint United Nations Programme on HIV and AIDS, 2020.

64. Kay M, Wijayanayaka S, Cook H, Hollingworth S. Understanding quality use of medicines in refugee communities in Australian primary care: a qualitative study. *Br J Gen Pract* 2016; **66**(647): e397-e409.

65. Kefale AT, Shebo HH. Availability of essential medicines and pharmaceutical inventory management practice at health centers of Adama town, Ethiopia. *BMC Health Serv Res* 2019; **19**(1): 254.

66. Kranzer K, Lawn SD, Meyer-Rath G, et al. Feasibility, yield, and cost of active tuberculosis case finding linked to a mobile HIV service in Cape Town, South Africa: a cross-sectional study. *PLoS Med* 2012; **9**(8): e1001281.

67. Kredo T, Adeniyi FB, Bateganya M, Pienaar ED. Task shifting from doctors to non‐doctors for initiation and maintenance of antiretroviral therapy. *Cochrane Database Syst Rev* 2014; (7): CD007331.

68. Kroon J, Prince E, Denicker GA. Trends in treatment performed in the Phelophepa Dental Clinic: 1995-2000. *SADJ* 2001; **56**(10): 462-6.

69. Labhardt ND, Motlomelo M, Cerutti B, et al. Home-based versus mobile clinic HIV testing and counseling in rural Lesotho: a cluster-randomized trial. *PLoS Med* 2014; **11**(12): e1001768.

70. Larson B, Schnippel K, Ndibongo B, et al. Rapid point-of-care CD4 testing at mobile HIV testing sites to increase linkage to care: an evaluation of a pilot program in South Africa. *J Acquir Immune Defic Syndr* 2012; **61**(2): e13-7.

71. Lewis M, Pettersson G. Governance in health care delivery: raising performance. Washington, DC: The World Bank; 2009.

72. Libamba E, Makombe S, Harries AD, et al. Scaling up antiretroviral therapy in Africa: learning from tuberculosis control programmes - the case of Malawi. *Int J Tuberc Lung Dis* 2005; **9**(10): 1062-71.

73. Masiye F, Chitah BM, McIntyre D. From targeted exemptions to user fee abolition in health care: experience from rural Zambia. *Soc Sci Med* 2010; **71**(4): 743-50.

74. Mavungu Landu DJ, Frédérich M, Manzambi Kuwekita J, et al. Quality of antimalarials in Kinshasa peri-urban areas with regard to local pharmaceutical legislation and regulation. *Int Health* 2019; **12**(4): 253-63.

75. Mc Donald T, Chapman RD, MacKenzie J. Primary health care rendered from mobile units: can computers help? *Curationis* 1994; **17**(2): 35-8.

76. McClintock HF, Alber JM, Schrauben SJ, Mazzola CM, Wiebe DJ. Constructing a measure of health literacy in sub-Saharan African countries. *Health Promot Int* 2019; **35**(5): 907-15.

77. McQuaid CF, McCreesh N, Read JM, et al. The potential impact of COVID-19-related disruption on tuberculosis burden. *Eur Respir J* 2020; **56**(2): 2001718.

78. Mhimbira FA, Cuevas LE, Dacombe R, Mkopi A, Sinclair D. Interventions to increase tuberculosis case detection at primary healthcare or community-level services. *Cochrane Database Syst Rev* 2017; **11**(11): CD011432.

79. Micah AE, Chen CS, Zlavog BS, Hashimi G, Chapin A, Dieleman JL. Trends and drivers of government health spending in sub-Saharan Africa, 1995–2015. *BMJ Glob Health* 2019; **4**(1): e001159.

80. Miyano S, Syakantu G, Komada K, Endo H, Sugishita T. Cost-effectiveness analysis of the national decentralization policy of antiretroviral treatment programme in Zambia. *Cost Eff Resour Alloc* 2017; **15**: 4.

81. Moore AF, Hawarden V. Discovery Digital Health strategy: COVID-19 accelerates online health care in South Africa. *EMCS* 2020; **10**(3): 1-18.

82. Moustakis J, Piperidis AA, Ogunrombi AB. The effect of COVID-19 on essential surgical admissions in South Africa: a retrospective observational analysis of admissions before and during lockdown at a tertiary healthcare complex. *S Afr Med J* 2020; **110**(9): 910-5.

83. Mubyazi GM, Magnussen P, Goodman C, et al. Implementing intermittent preventive treatment for malaria in pregnancy: review of prospects, achievements, challenges and agenda for research. *Open Trop Med J* 2008; **1**: 92-100.

84. Mufusama J-P, Ndjoko Ioset K, Feineis D, Hoellein L, Holzgrabe U, Bringmann G. Quality of the antimalarial medicine artemether – lumefantrine in 8 cities of the Democratic Republic of the Congo. *Drug Test Anal* 2018; **10**(10): 1599-606.

85. Mwambete KD. Irrational antibiotic usage in boarding secondary school settings in Dar es Salaam. *East Afr J Public Health* 2009; **6**(2): 200-4.

86. Tunisia: rural contraceptive distribution project has impact. *Netw Int Fertil Res Program* 1979.

87. Nayyar GML, Breman JG, Newton PN, Herrington J. Poor-quality antimalarial drugs in southeast Asia and sub-Saharan Africa. *Lancet Infect Dis* 2012; **12**(6): 488-96.

88. Nguyen HT, Torbica A, Brenner S, et al. Economic evaluation of user-fee exemption policies for maternal healthcare in Burkina Faso: evidence from a cost-effectiveness analysis. *Value Health* 2020; **23**(3): 300-8.

89. Nyato D, Kuringe E, Drake M, et al. Participants' accrual and delivery of HIV prevention interventions among men who have sex with men in sub-Saharan Africa: a systematic review. *BMC Public Health* 2018; **18**(1): 370.

90. O’Doherty D, Dromey M, Lougheed J, Hannigan A, Last J, McGrath D. Barriers and solutions to online learning in medical education–an integrative review. *BMC Med Educ* 2018; **18**(1): 130.

91. Onakpoya IJ, Heneghan CJ, Aronson JK. Delays in the post-marketing withdrawal of drugs to which deaths have been attributed: a systematic investigation and analysis. *BMC Med* 2015; **13**(1): 26.

92. Onwujekwe O, Agwu P, Orjiakor C, et al. Corruption in Anglophone West Africa health systems: a systematic review of its different variants and the factors that sustain them. *Health Policy Plan* 2019; **34**(7): 529-43.

93. Organisation for Economic Co-operation and Development. Pharmaceutical innovation and access to medicines. Paris: Organisation for Economic Co-operation and Development; 2018.

94. Oualla K, Nouiakh L, Acharfi N, et al. How Is Morocco reacting to COVID-19 crisis in anticancer centers? *Cancer Control* 2020; **27**(3): 1073274820941973.

95. Palmer JJ, Gilbert A, Choy M, Blanchet K. Circumventing 'free care' and 'shouting louder': using a health systems approach to study eye health system sustainability in government and mission facilities of north-west Tanzania. *Health Res Policy Syst* 2016; **14**(1): 68.

96. Parmar D, Banerjee A. How do supply- and demand-side interventions influence equity in healthcare utilisation? Evidence from maternal healthcare in Senegal. *Soc Sci Med* 2019; **241**: 112582.

97. Pathmanathan I, Ahmedov S, Pevzner E, et al. TB preventive therapy for people living with HIV: key considerations for scale-up in resource-limited settings. *Int J Tuberc Lung Dis* 2018; **22**(6): 596-605.

98. Pheage T. Dying from lack of medicines. *Africa Renewal* 2017; **30**(3): 24–5.

99. Pillay L, Janse van Rensburg DCC, Jansen van Rensburg A, et al. Nowhere to hide: the significant impact of coronavirus disease 2019 (COVID-19) measures on elite and semi-elite South African athletes. *J Sci Med Sport* 2020; **23**(7): 670-9.

100. Ponsar F, Tayler-Smith K, Philips M, et al. No cash, no care: how user fees endanger health—lessons learnt regarding financial barriers to healthcare services in Burundi, Sierra Leone, Democratic Republic of Congo, Chad, Haiti and Mali. *Int Health* 2011; **3**(2): 91-100.

101. Ravit M, Audibert M, Ridde V, De Loenzien M, Schantz C, Dumont A. Do free caesarean section policies increase inequalities in Benin and Mali? *Int J Equity Health* 2018; **17**(1): 71.

102. Ravit M, Audibert M, Ridde V, De Loenzien M, Schantz C, Dumont A. Removing user fees to improve access to caesarean delivery: a quasi-experimental evaluation in western Africa. *BMJ Glob Health* 2018; **3**(1): e000558.

103. Ridde V, Agier I, Jahn A, et al. The impact of user fee removal policies on household out-of-pocket spending: evidence against the inverse equity hypothesis from a population based study in Burkina Faso. *Eur J Health Econ* 2015; **16**(1): 55-64.

104. Robert E, Samb OM, Marchal B, Ridde V. Building a middle-range theory of free public healthcare seeking in sub-Saharan Africa: a realist review. *Health Policy Plan* 2017; **32**(7): 1002-14.

105. Roberton T, Carter ED, Chou VB, et al. Early estimates of the indirect effects of the COVID-19 pandemic on maternal and child mortality in low-income and middle-income countries: a modelling study. *Lancet Glob Health* 2020; **8**(7): e901-e8.

106. Roth L, Bempong D, Babigumira JB, et al. Expanding global access to essential medicines: investment priorities for sustainably strengthening medical product regulatory systems. *Global Health* 2018; **14**(1): 102.

107. Sagaon‐Teyssier L, Singh S, Dongmo‐Nguimfack B, Moatti JP. Affordability of adult HIV/AIDS treatment in developing countries: modelling price determinants for a better insight of the market functioning. *J Int AIDS Soc* 2016; **19**(1): 20619.

108. Semo BW, Frissa SM. The mental health impact of the covid-19 pandemic: implications for sub-saharan africa. *Psychol Res Behav Manage* 2020; **13**: 713-20.

109. Sherrard-Smith E, Hogan AB, Hamlet A, et al. The potential public health consequences of COVID-19 on malaria in Africa. *Nat Med* 2020; **26**(9): 1411-6.

110. Siedner MJ, Kraemer JD, Meyer MJ, et al. Access to primary healthcare during lockdown measures for COVID-19 in rural South Africa: an interrupted time series analysis. *BMJ Open* 2020; **10**: e043763.

111. Smith P, Tolla T, Marcus R, Bekker L-G. Mobile sexual health services for adolescents: investigating the acceptability of youth-directed mobile clinic services in Cape Town, South Africa. *BMC Health Serv Res* 2019; **19**(1): 584.

112. Stadler J, Delany S. The 'healthy brothel': the context of clinical services for sex workers in Hillbrow, South Africa. *Cult Health Sex* 2006; **8**(5): 451-63.

113. Stormacq C, Wosinski J, Boillat E, Van den Broucke S. Effects of health literacy interventions on health-related outcomes in socioeconomically disadvantaged adults living in the community: a systematic review. *JBI Evid Synth* 2020; **18**(7): 1389-469.

114. Suleman S, Woliyi A, Woldemichael K, et al. Pharmaceutical regulatory framework in Ethiopia: a critical evaluation of its legal basis and implementation. *Ethiop J Health Sci* 2016; **26**(3): 259-76.

115. Surie D, Interrante JD, Pathmanathan I, et al. Policies, practices and barriers to implementing tuberculosis preventive treatment-35 countries, 2017. *Int J Tuberc Lung Dis* 2019; **23**(12): 1308-13.

116. Taylor C, Griffiths F, Lilford R. Affordability of comprehensive community health worker programmes in rural sub-Saharan Africa. *BMJ Glob Health* 2017; **2**(3): e000391.

117. Teachout M, Zipfel C. The economic impact of COVID-19 lockdowns in sub-Saharan Africa. London: International Growth Centre, 2020.

118. The World Bank. Global economic prospects. Washington, DC: The World Bank, 2020.

119. Tivura M, Asante I, van Wyk A, et al. Quality of artemisinin-based combination therapy for malaria found in Ghanaian markets and public health implications of their use. *BMC Pharmacol Toxicol* 2016; **17**(1): 48.

120. Togun T, Kampmann B, Stoker NG, Lipman M. Anticipating the impact of the COVID-19 pandemic on TB patients and TB control programmes. *Ann Clin Microbiol Antimicrob* 2020; **19**(1): 21.

121. United Nations Economic Commission for Africa. ECA estimates billions worth of losses in Africa due to COVID-19 impact. Addis Ababa: United Nations Economic Commission for Africa, 2020.

122. United Nations Secretary-General's High-Level Panel on Access to Medicines. Strengthening local pharmaceutical production in Africa to improve and sustain Access to Medicines. New York: United Nations Secretary-General's High-Level Panel on Access to Medicines, 2016.

123. Guetiya Wadoum RE, Samin A, Mafopa NG, et al. Mobile health clinic for the medical management of clinical sequelae experienced by survivors of the 2013-2016 Ebola virus disease outbreak in Sierra Leone, West Africa. *Eur J Clin Microbiol Infect Dis* 2017; **36**(11): 2193-200.

124. Walker G. Primary health care in Botswana: a study in cost-effectiveness. *J R Soc Med* 1976; **69**(12): 936-9.

125. Walker G, Gish O. Mobile health services: a study in cost-effectiveness. *Med Care* 1977; **15**(4): 267-76.

126. Watson SI, Sahota H, Taylor CA, Chen Y-F, Lilford RJ. Cost-effectiveness of health care service delivery interventions in low and middle income countries: a systematic review. *Glob Health Res Policy* 2018; **3**(1): 17.

127. Weiss DJ, Bertozzi-Villa A, Rumisha SF, et al. Indirect effects of the COVID-19 pandemic on malaria intervention coverage, morbidity, and mortality in Africa: a geospatial modelling analysis. *Lancet Infect Dis* 2020; **21**(1): 59-69.

128. White RO, Eden S, Wallston KA, et al. Health communication, self-care, and treatment satisfaction among low-income diabetes patients in a public health setting. *Patient Educ Couns* 2015; **98**(2): 144-9.

129. Wilkinson D, Sach ME. Cost effective on-site screening for anaemia in pregnancy in primary care clinics. *S Afr Med J* 1997; **87**(4): 463-5.

130. Wirtz VJ, Hogerzeil HV, Gray AL, et al. Essential medicines for universal health coverage. *Lancet* 2017; **389**(10067): 403-76.

131. World Health Organization. COVID-19 Supply Chain System: requesting and receiving supplies. Geneva: World Health Organization, 2020.

132. World Health Organization. The potential impact of health service disruptions on the burden of malaria: a modelling analysis for countries in sub-Saharan Africa. Geneva: World Health Organization, 2020.

133. World Trade Organization. Waiver from certain provisions of the TRIPS agreement for the prevention, containment and treatment of COVID-19: communication from India and South Africa. Geneva: World Trade Organization, 2020.

134. Ziadé N, el Kibbi L, Hmamouchi I, et al. Impact of the COVID-19 pandemic on patients with chronic rheumatic diseases: a study in 15 Arab countries. *Int J Rheum Dis* 2020; **23**(11): 1550-7.
